# *In silico* model development and optimization of *in vitro* lung cell population growth

**DOI:** 10.1371/journal.pone.0300902

**Published:** 2024-05-15

**Authors:** Amirmahdi Mostofinejad, David A. Romero, Dana Brinson, Alba E. Marin-Araujo, Aimy Bazylak, Thomas K. Waddell, Siba Haykal, Golnaz Karoubi, Cristina H. Amon

**Affiliations:** 1 Department of Mechanical and Industrial Engineering, University of Toronto, Toronto, Ontario, Canada; 2 Institute of Biomedical Engineering, University of Toronto, Toronto, Ontario, Canada; 3 Latner Research Laboratories, Division of Thoracic Surgery, University Health Network, Toronto, Ontario, Canada; 4 Division of Plastic Surgery, University Health Network, Toronto, Ontario, Canada; REVA University, INDIA

## Abstract

Tissue engineering predominantly relies on trial and error *in vitro* and *ex vivo* experiments to develop protocols and bioreactors to generate functional tissues. As an alternative, *in silico* methods have the potential to significantly reduce the timelines and costs of experimental programs for tissue engineering. In this paper, we propose a methodology to formulate, select, calibrate, and test mathematical models to predict cell population growth as a function of the biochemical environment and to design optimal experimental protocols for model inference of *in silico* model parameters. We systematically combine methods from the experimental design, mathematical statistics, and optimization literature to develop unique and explainable mathematical models for cell population dynamics. The proposed methodology is applied to the development of this first published model for a population of the airway-relevant bronchio-alveolar epithelial (BEAS-2B) cell line as a function of the concentration of metabolic-related biochemical substrates. The resulting model is a system of ordinary differential equations that predict the temporal dynamics of BEAS-2B cell populations as a function of the initial seeded cell population and the glucose, oxygen, and lactate concentrations in the growth media, using seven parameters rigorously inferred from optimally designed *in vitro* experiments.

## 1 Introduction

Tissue engineering is a subfield of biomedical engineering that aims to construct functional tissues and organs using engineering principles applied to biological systems. This field has long relied on rigorous *in vitro*, *ex vivo*, and *in vivo* empirical studies to identify culture conditions that achieve experimental objectives, such as maximizing cell yield/function or minimize variability, among others [1]. Once optimal culture conditions have been identified, bioreactor devices and associated protocols can be designed to consistently and economically achieve them.

*In silico* approaches, i.e., the use of mathematical models and relevant computational tools, have become a new paradigm in bioengineering and biomedical studies over the last few years [2]. In tissue engineering applications, *in silico* approaches help researchers to formulate experimentally testable hypotheses for how the cells behave with quantitative and qualitative predictions about cell populations and population ratios [3]. In addition, mathematical models can improve our understanding of complex biological phenomena, guide experimental studies, and support the design and optimization of bioreactors and associated experimental protocols [4] for scaled-up production of functional biological tissues. Notably, using mathematical models for tissue engineering applications allows researchers to leverage advances in Simulation-based Design & Optimization (SBDO), a diverse collection of methods and practices for the efficient, systematic use of computer and physical models to support the design and optimization of engineering systems. SBDO has the potential to significantly advance tissue engineering by accelerating discovery and by reducing the time and cost required for the development of next-generation bioreactor devices and experimental protocols [5, 6].

Several mathematical models have been proposed for neotissue growth focusing on bone [7, 8], cartilage [9], and neural tissues [10, 11]. Most of these models consist of a system of ordinary and partial differential equations for the cell population and biochemical concentrations [4, 8, 10–12], while others also consider mechanical cues such as shear stress [7, 9, 13]. These differential equations include coefficients and mathematical expressions representing different aspects of system dynamics, such as diffusion rates, chemical reactions, and modulating effects of biochemical and mechanical cues, all of which must be calibrated and validated.

Due to data availability, cost, and timeline constraints, not all the mathematical models found in the literature have been calibrated and/or validated based on specially designed *in vitro* or *ex vivo* experiments [9]. Instead, researchers may rely on model parameters estimated under different experimental contexts [7], or calibrate their models to reproduce qualitative behavior only [14]. Additionally, in most cases, the proposed models have not been analyzed for uniqueness or identifiability [7, 8, 10, 11, 13]. Without this, the estimated subset of model parameters is not guaranteed to be physically consistent [12, 15] and generalizable.

One of the potential application areas of the SBDO paradigm is the engineering of functional lung and airway tissues, organoids, and even whole organs, with potential applications in screening and development of drug therapies [16], disease modeling [17] and, eventually, to create *de novo* tissues and organs for human transplantation [18]. A large body of work has focused on engineering airway tissues using synthetic [19–21] and donor-derived scaffolds [22, 23]. Among the many scientific and translational challenges of this line of research, one of the most significant is the design and optimization of protocols and devices to sustain the relevant cells as they deposit and attach to suitable scaffolds, proliferate, migrate, and differentiate into the targeted cell types required for functional tissues. A promising approach focuses on creating scaffolds through partial or total decellularization of donor organs. This strategy can result in 3D scaffolds that already have the necessary biochemical and mechanical cues needed for subsequent recellularization [22, 24] with recipient-derived adult cells [24], embryonic stem cells [25], or induced pluripotent stem cells (iPSC) [26, 27]. Still heavily under research, several airway-relevant cell lines are used instead of donor cells for both accelerating discovery and proof-of-concept studies, including BEAS-2Bs [28–30], A549s [31], Calu-3s [32], and human tracheal epithelial cells [33]. Despite the utility of these cell types, no mathematical models currently exist to describe their population dynamics under targeted culture conditions *in vitro* and/or *ex vivo*. The availability of such mathematical models can help accelerate discovery and translation in tissue engineering.

In this work, we propose a detailed, rigorous methodology for developing *in silico* models for neotissue growth *in vitro* and *ex vivo*. We propose a model-based design of experimental protocols (MBDEP) approach that, leveraging these *in silico* models, defines the spatio-temporal sampling frequency required to optimally infer the model parameters based on experimental data. Starting from a set of biologically-informed model proposals, the proposed model development methodology uniquely combines methods from model inference (e.g., non-linear regression), model selection (data splitting), design of experiments, mathematical statistics (e.g., identifiability analysis [15, 34]), sensitivity analysis [35]), and optimization.

As a case study to illustrate our model development approach, this paper describes the development of the first mathematical model capable of predicting the population dynamics of bronchio-alveolar epithelial cells (BEAS-2Bs). BEAS-2Bs are non-cancer, immortalized cells that grow in monolayers [36], and replace normal human bronchial epithelial cells as a model in various toxicology studies [37]. BEAS-2Bs were chosen to showcase the proposed methodology because they are often used in tissue engineering, organ regeneration, and transplantation studies, including decellularization and recellularization of airway tissue scaffolds [24, 38, 39], due to their ability to grow in a lab setting and mimic the function of the human airway epithelium [29, 37]. Thus, the mathematical model developed in this paper will be directly applicable to these contexts. Importantly, the proposed model development methodology is directly applicable to the formulation, calibration and validation of mathematical models for any other single cell line population and, with a suitable family of model proposals, to multicellular population dynamics.

## 2 Materials and methods

### 2.1 Experimental setup

#### Cell culture

*In vitro* experiments are run for five days with four initial cell populations of 25,000, 50,000, 100,000, and 200,000 cells/well in 6-well plates. Each replicate has three wells seeded with the specified population of BEAS-2Bs [28, 37]. Each experiment has three replicates (3 × 3 = 9 total wells per initial cell density). The experiments did not involve any media change to observe the effect of more extreme concentrations. We ran four experiments at different glucose and oxygen levels to see their effects on cell population dynamics. For the first experiment, the media was 3 mL Dulbecco’s Modified Eagle Medium (DMEM, Gibco, USA) with high glucose and pyruvate, and the cells were cultured at 37°C, normoxic incubator [40] (18.6% oxygen), with 5% carbon dioxide. The second experiment used the same configuration as experiment one with DMEM with low glucose and pyruvate as the culture medium. For the third and fourth experiments, we altered experiments one and two by culturing the cells at 37°C in the tri-gas incubator [41], CellXpert C170i (Eppendorf, Germany), with 5% oxygen and 5% carbon dioxide.

#### Measurements

In each experiment, we removed the plates from the respective incubator and took 200 *μ*L media samples and measured glucose, lactate, oxygen, potassium, sodium, and calcium concentrations using RAPIDPoint 500 Blood Gas Systems (Siemens Healthcare Limited, Canada) six hours after seeding and then every 12 hours (Experiment 1), or 24 hours (Experiments 2, 3, and 4), with the last measurement taken at 114 hours (5 days). At the same intervals, we took five images of the wells (EVOS FL Cell Imaging System) to estimate the total cell count.

### 2.2 Model development methodology

**Fig 1:**
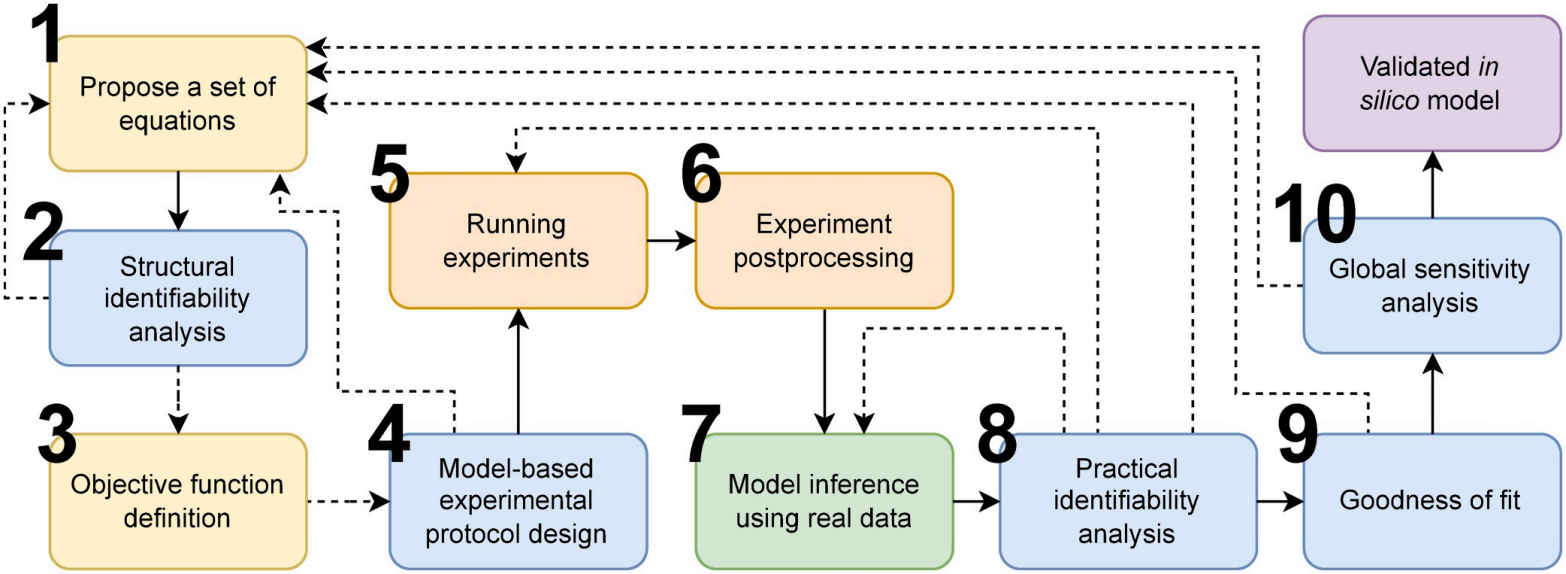
Model development framework. The green box shows the mathematical model development steps, blue boxes depict the mathematical checks on the model, orange boxes display the experimental steps, and yellow boxes show the initial steps in model development.

This paper proposes a methodology for the development of mathematical models for neotissue growth dynamics. The overarching problem here is to identify, calibrate and validate a mathematical (*in silico*) model for the population dynamics of a given cell type as influenced by a pre-defined set of biochemical stimuli. Sections 2.2.2 to 2.2.10 below discuss in more detail each of the corresponding steps of the proposed methodology, shown in Fig 1. Results of applying this methodology for *in silico* modeling of cell population dynamics during in vitro culture of BEAS-2Bs cells are presented in Sec. 3.

First, we start with designing a model for cell culture in well plates under static (no flow) conditions with different biochemical environments. This model will focus on the effect of chemical substrates (external stimuli) on proliferation and apoptosis rates (cellular responses). The model proposals are informed by the known biology and physics of the system. After creating a library of candidate models that govern the dynamics, the models are studied through structural identifiability analysis (section 2.2.2). The structurally identifiable models are considered for the next steps of model development. The next step is to define the objective function that encodes the model inference goal, typically minimizing the prediction error of the model with respect to the experimental data. Alternative goals may be formulated as part of the objective function or as optimization constraints, e.g., minimizing the variance of the parameter estimates, minimizing the number of parameters to be estimated, or minimizing residuals with respect to physical or empirical laws, among others. Then, an experimental protocol for data collection (e.g., sampling frequency) is designed so that it is optimal for the family of model proposals over a range of assumed noise levels. Once the experiments are conducted and the data collected and post-processed, the methodology focuses on model inference, i.e., model calibration (fitting) and selection. The result of this process is a single model selected from the set of model proposals that best fits the data according to the previously defined objective function. Next, practical identifiability analysis confirms that the inferred model parameters are unique and finite, i.e., that the objective function has a single global optimum. Then, the goodness of fit of the model is quantified (model validation) to provide an estimate of the expected predictive performance of the model under experimental conditions that are different from those used during model calibration and selection. We conclude the model development procedure using global sensitivity analysis to rank the controllable experimental parameters according to their predicted effect on the cell population. Sensitivity analysis is also used as a diagnostic tool for model calibration by identifying the subset of model parameters that have the greatest influence on model fit.

#### 2.2.1 Model proposals

In this work, we will discuss the modeling methodology focusing only on the dynamics of the cell population, including proliferation and apoptosis of a single cell line in a biochemical environment that is governed by advection, diffusion, and reaction phenomena. However, the methodology can be applied for inference of mathematical models, including other aspects of neotissue growth, such as scaffold biomechanics, cell-cell, and cell-scaffold interactions, cell migration via chemotaxis and haptotaxis, and cell differentiation, by including additional equations codifying the underlying physics [42].

Using the mass balance equation for cell density and for the chemical species concentrations (also referred to as “substrates” in the literature) leads to a set of coupled advection-diffusion-reaction equations that describe the spatiotemporal cells densities and substrates concentrations fields [43]. These spatiotemporal equations relate the rate of change of specific concentrations and densities to the diffusion, advection, and reaction rates. The equations are defined as,

$$\begin{array}{c} \begin{array}{cc} \frac{\partial c_{i}}{\partial t}\left(X,t\right) & +\nabla .\left(c_{i}\left(X,t\right)u\left(X,t\right)\right)-\nabla .\left(D_{c_{i}}\nabla c_{i}\left(X,t\right)\right)\\ & =M_{c_{i}}\left(N\left(X,t\right),C\left(X,t\right)\right), \end{array} \end{array}$$ (1)

$$\begin{array}{c} \begin{array}{cc} \frac{\partial n_{j}}{\partial t}\left(X,t\right) & +\nabla .\left(n_{j}\left(X,t\right)u\left(X,t\right)\right)-\nabla .\left(D_{n_{j}}\nabla n_{j}\left(X,t\right)\right)\\ & =M_{n_{j}}\left(N\left(X,t\right),C\left(X,t\right)\right). \end{array} \end{array}$$ (2)

Here, *c*_*i*_ is concentration, and *n*_*j*_ is density for each chemical species, *i*, or cell line, *j*, respectively. $D_{c_{i}}$ and $D_{n_{j}}$ are the diffusion coefficients, **u** is the fluid velocity, $M_{c_{i}}$ is the substrate reaction rate, $M_{n_{j}}$ is the cellular responses (proliferation, death, and differentiation), **X** is the position vector, *t* is time, **C** and **N** are vectors containing all concentrations and densities, respectively, and ∇⋅ is the divergence operator.

The general governing Eqs 3 and 4 have been applied to a broad spectrum of growth and transport of biological processes [44–46]. Simplified versions of Eqs 1 and 2 can be obtained when the concentration and cell density fields are spatially homogeneous, such as in the case of *in vitro* submerged static cultures on well plates. Since there is no spatial variability, the diffusion and advection terms in Eqs 1 and 2 can be disregarded, thus

$$\begin{array}{c} \frac{\text{d}c_{i}}{\text{d}t}\left(t\right)=M_{c_{i}}\left(N\left(t\right),C\left(t\right)\right), \end{array}$$ (3)

$$\begin{array}{c} \frac{\text{d}n_{j}}{\text{d}t}\left(t\right)=M_{n_{j}}\left(N\left(t\right),C\left(t\right)\right). \end{array}$$ (4)

Eqs 3 and 4 show that the rate of change for concentrations and cell densities are defined by the reaction and response rates in a homogeneous domain. These rates are the summation of the production and consumption rates in the case of chemical species concentrations, and the summation of proliferation, death, and differentiation rates in the case of cell line densities [8, 10, 47].

Let us now look at specific, biologically informed functional forms for the right hand side of Eqs 3 and 4. For neotissues consisting of a single cell line that does not differentiate, we propose a model for the growth of the cell population incorporating proliferation and apoptosis rates [42] as,

$$\begin{array}{c} \frac{\text{d}n}{\text{d}t}\left(t\right)=\overset{\text{proliferation}\;\text{rate}}{\overset{︷}{\underset{\text{effect}\;\text{of}\;\text{the}\;\text{environment}\;(F_{\text{env}})}{\underset{︸}{f_{1}\left(c_{o}\left(t\right)\right)f_{2}\left(c_{g}\left(t\right)\right)f_{3}\left(c_{l}\left(t\right)\right)}}\underset{\text{growth}\;\text{limited}\;\text{by}\;\text{space}}{\underset{︸}{\beta n\left(t\right)(1-\frac{n(t)}{n_{\text{max}}})}}}}-\overset{\text{death}\;\text{rate}}{\overset{︷}{\delta n(t)}} \end{array}$$ (5)

Here, *n*, *c*_*o*_, *c*_*g*_, *c*_*l*_, *n*_max_, *δ*, and *β* are the cell density, concentrations of oxygen, glucose, and lactate, maximum cell density, apoptosis rate constant, and coefficient of proliferation, respectively. As seen in the equation, it consists of two terms, one for the proliferation rate and the other for the death rate. The proliferation rate term is proportionally modulated by two effects [48], the first shows the biochemical stimuli effect, and the other shows the proliferation rate limited by the physical space. A logistic function for the latter term shows that the growth rate per capita linearly drops as the population increases until cell population saturation [49, 50].

In Eq 5, the potential modulating effect of the biochemical substrates is usually defined in the literature as,

$$\begin{array}{cc} f_{k}\left(c_{i}\left(t\right)\right)=1, & \text{zero-order}, \end{array}$$ (6a)

$$\begin{array}{cc} f_{k}\left(c_{i}\left(t\right)\right)=c_{i}\left(t\right), & \text{first-order}, \end{array}$$ (6b)

$$\begin{array}{cc} f_{k}\left(c_{i}\left(t\right)\right)=\frac{c_{i}\left(t\right)}{c_{i}\left(t\right)+K_{i}}, & \text{MMK}\;\text{(positive}\;\text{feedback)}, \end{array}$$ (6c)

$$\begin{array}{cc} f_{k}\left(c_{i}\left(t\right)\right)=\frac{K_{i}}{c_{i}\left(t\right)+K_{i}}, & \text{MMK}\;\text{(negative}\;\text{feedback)},\\ & \left(i,k\right)\in \{(o,1),(g,2),(l,3)\}, \end{array}$$ (6d)

where *K*_*i*_ is the Michaelis-Menten Kinetics (MMK) constant. Each of the three substrates can either have a zero-order [8], linear [10], or Michaelis-Menten [7, 47] effect on cell growth rate. The modulating effects depend on the cell type and the chemical substrate under consideration and, when unknown, can be the subject of data-driven model selection methods. It is important to note that, depending on the specific cell type, additional chemical substrates may have significant modulating effects on the cell population, e.g., growth factors, blockers and inhibitors, among others. These can be trivially included in the model through additional modulating terms in Eq 4 and additional advection-diffusion-reaction equations (Eq 1).

For the purpose of describing the methodology and illustrating it with a specific cell line, we will limit our discussion to the modulating effects of glucose, oxygen, and lactate. These nutrients affect growth the most and play a crucial role in tissue viability [7, 47, 51]. The reaction rates in Eqs 3 and 4 are dependent on concentrations and neotissue density *n* as,

$$\begin{array}{c} M_{i}\left(n,c_{i},c_{i^{\prime }}\right)=R\left(n\right)R_{i}\left(c_{i},c_{i^{\prime }}\right)=nR_{i}\left(c_{i},c_{i^{\prime }}\right), \end{array}$$ (7)

where *i*′ is the inhibitor and *R*_*i*_(*c*_*i*_, *c*_*i*′_) is referred to as the reaction term and can take one of several mathematical forms, each representing different kinetics [10, 42, 52],

$$\begin{array}{cc} R_{i}\left(c_{i},c_{i^{\prime }}\right)=V_{i}, & \text{zero-order},\\ R_{i}\left(c_{i},c_{i^{\prime }}\right)=V_{i}c_{i}, & \text{first-order},\\ R_{i}\left(c_{i},c_{i^{\prime }}\right)=\frac{V_{i}c_{i}}{c_{i}+{\bar{c}}_{i}}, & \text{Michaelis-Menten},\\ R_{i}\left(c_{i},c_{i^{\prime }}\right)=\frac{V_{i}c_{i}}{c_{i}+{\bar{c}}_{ii^{\prime }}c_{i^{\prime }}+{\bar{c}}_{i^{\prime }}}, & \text{competitive}\;\text{inhibition}. \end{array}$$ (8)

Here, *V*_*i*_, ${\bar{c}}_{i}$, ${\bar{c}}_{ii^{\prime }}$, are the reaction constant, the MMK constant, and the inhibition constant, respectively. With a set of biologically-informed mathematical models, model calibration and selection methods utilizing the experimental observations will find the best kinetics for each substrate and its corresponding parameters (Eqs 6 and 8).

The proposed mass-conservation-based neotissue growth model treats the entire population of cells as homogeneous in their density and type without considering multiple cell types, their interactions, and cell transitions from one type to another (i.e., cell differentiation). However, the framework we propose can be easily applied to multiple cell types by adding additional equations similar to Eq 2 for each type, and including transitions between cell types through the response terms. Future spatiotemporal (nonhomogeneous) models will include diffusion and advection terms for the cell population similar to Eqs 1 and 2 [53].

#### 2.2.2 Structural identifiability analysis

Mathematical models in biology are usually defined with differential equations as [15],

$$\begin{array}{c} \dot{Y}=f\left(Y,\Theta ,u\right). \end{array}$$ (9)

In this equation, **Y** is a vector containing a set of state variables, e.g., substrate concentration and cell densities, **Θ** is a vector of model parameters, **u** is a vector of the external stimuli, and $\dot{Y}$ is the rate of change of the state variables over time. Usually, not all the state variables can be measured directly during the experiments; thus, the observables, **z** are denoted as,

$$\begin{array}{c} z=g(Y,\Theta ,u). \end{array}$$ (10)

A mathematical model is identifiable whenever a unique set of observations or measurements would result in one and only one set of model parameters [50]. Mathematically, if **Θ** and **Φ** are two valid sets of model parameters, then

$$\begin{array}{c} g(Y,\Theta ,u)=g(Y,\Phi ,u)\;\Rightarrow \;\Theta =\Phi . \end{array}$$ (11)

Structural identifiability analysis is performed on the mathematical model before model calibration, and it focuses on the relation between the state variables and observables. The model is discarded or modified if it is deemed that data collected about the observables, regardless of the amount of data, will not result in a unique set of model parameters. If a model is structurally identifiable, all the model parameters can be estimated from a sufficiently large number of observable measurements [4, 54]. To make this determination, the analysis consists of creating a modified differential algebraic equation (DAE) form from model equations that meets a certain rank criterion. The observability matrix is then obtained via symbolic techniques. The model is considered structurally identifiable if the observability matrix has full rank [55].

#### 2.2.3 Objective function definition

Generally, inferring a parametric model from data is a mathematical optimization problem, in which a set of model parameters is estimated that maximize a user-defined measure of ‘goodness of fit’. Two standard methods for parameter estimation of mathematical models are maximum likelihood estimation (MLE) and nonlinear least squares (NLS) [56], which use the likelihood function and the sum of squared errors as objective functions, respectively. The choice of objective function consolidates the assumptions about the data into the calibration process. In MLE, the posterior probability of the observed data is maximized based on the known or assumed statistical distribution of the data. In contrast, NLS minimizes the sum of squared residuals between observations and predictions (NLS) without any distributional assumption about the data.

In this work, we employ MLE because it produces a suitable frequentist formulation of the calibration process without imposing unwarranted assumptions about the data and noise distribution. The likelihood function for independent and identically distributed observations (i.i.d.), *Z*_*i*_, is defined as the multiplication of their probability functions (*p*) as,

$$\begin{array}{c} L\left(\Theta ;Z\right)=\Pi_{i=1}^{n}\;p\left(\Theta ;z_{i}\right). \end{array}$$ (12)

Let us assume that the observations are normally distributed, with a time-dependent but unknown mean as predicted by the model with unknown parameters **Θ** and unknown standard deviation. Then, minimizing the negative log-likelihood objective function, −*ℓ*(**Θ**;**Z**), is mathematically equivalent to the weighted NLS problem defined as [4],

$$\begin{array}{c} \text{LS}=-\ell \left(\Theta ;Z\right)=\frac{1}{2}\sum_{j=1}^{n_{z}}\sum_{k=1}^{n_{k}}w_{j}\left(t_{k}\right){\left(Z_{j}\left(t_{k}\right)-z_{j}\left(t_{k},\Theta \right)\right)}^{2} \end{array}$$ (13)

where $w_{j}\left(t_{k}\right)=\sigma_{j}^{-2}\left(t_{k}\right)$. Here, *n*_*k*_, *n*_*z*_, *z*_*j*_, *Z*_*j*_, and *σ*_*j*_(*t*_*k*_) are, respectively, the number of time points, the number of observables, the model predictions, and the mean and standard deviation of each observable at each time point across experimental replicates, respectively.

#### 2.2.4 Model-based design of experimental protocols (MBDEP)

We propose an approach to design the data collection protocol so that it minimizes the error of estimated model parameters for a given set of model proposals under expected experimental noise levels. Given the non-linearity of the models we use here, our approach is based on statistical simulation, as follows.

First, we identify a set of plausible model parameters, e.g., taking parameter values from the relevant literature, similar experiments with other cell types, or other experiments done with the cell type of interest. Alternatively, some model parameters may be roughly estimated based on the known biology and physics of the process. Next, we do a forward modeling step, in which we use the models with these assumed parameter values to generate simulated noiseless experimental data about the observables. Gaussian noise is then added to this data, i.e.,

$$\begin{array}{c} Z_{j}\left(t_{k}\right)=z_{j}\left(t_{k}\right)\left(1+\epsilon \eta_{i}\left(t_{k}\right)\right)\\ \eta_{j}\left(t_{k}\right)∼N(\mu =0,\sigma^{2}=1) \end{array}$$ (14)

where, *ϵ* is the noise level, *z*_*j*_(*t*_*k*_) represents the simulated value of observable *j* at time *t*_*k*_, and N is the Gaussian probability distribution function. Different noise levels, *ϵ* ∈ {0, 0.05, 0.1, 0.2, 0.3, 0.4, 0.5}, can be used for this analysis to observe how different experimental noise levels would affect model inference. Note that here we assume that all observables can be measured with a similar level of experimental error (noise). However, the same approach can be used with different noise levels for each observable, e.g., representing the availability of different measurement techniques or equipment with different levels of accuracy. The simulated data with added noise is then used for MLE or NLS estimation of model parameters (Sec. 2.2.7), and the difference between the assumed and the estimated values of the model parameters is calculated.

These steps are repeated with multiple sets of simulated data and different spatio-temporal sampling frequencies (i.e., with different amounts of data). The results of this effort allow us to identify the sampling frequencies that are required to ensure that the estimation of model parameters is robust to the expected levels of experimental noise. Furthermore, if there are external constraints on the experimental procedure (e.g., equipment/personnel availability, cost, timelines, etc.), the proposed MBDEP can be used to check the feasibility of model inference and thus signal the need for reformulating experimental goals.

#### 2.2.7 Model inference

Once the experimental data has been collected, the data is split into three subsets for calibration (60% of the data), selection (20%), and validation (20%), following best practices. The calibration data set is then used to formulate, for each candidate model, a nonlinear optimization problem to determine the set of model parameters that best fit the data, i.e.,

$$\begin{array}{c} \Theta^{*}=\text{arg}\;\underset{\Theta }{\text{min}}-\ell \left(\Theta ;Z\right) \end{array}$$ (15)

The optimization problem posed in Eq 15 is solved through a multi-start strategy, which helps with detecting and averting the local minima [57]. A maxi-min Latin hypercube method is utilized to generate a set of initial guesses that cover the search space with guaranteed lower-dimension projection properties [58, 59]. Starting from each initial parameter guess, the optimization problem is solved using the Adam stochastic gradient decent-based method [60]. Then, the best-performing solutions are used as starting points for a further optimization stage using the BFGS method [61] to ensure final convergence.

Model selection is made between the candidate models by checking how well they match the time evolution of state variables, i.e., cell populations and biochemical concentrations on the selection dataset. Several error metrics (e.g., MSE, RMSE) can be used for this purpose. However, given that the candidate models may have different numbers of parameters, in this work, we use the Akaike Information Criterion (AIC) or Bayesian Information Criterion (BIC),

$$\begin{array}{c} \text{AIC}=2k-2\ell (\Theta ;Z),\\ \text{BIC}=k\;\text{log}\left(m_{s}\right)-2\ell \left(\Theta ;Z\right). \end{array}$$ (16)

to select the model that best balances model complexity and goodness of fit [4, 62, 63]. In Eq 16, log(*x*) is the natural logarithm of *x*, *k* is the number of inferred parameters, and *m*_*s*_ is the number of observations in the selection dataset. The use of AIC and BIC has been widely discussed in the literature, and although no definitive recommendations have been made, it is commonly accepted that AIC is an optimal rule for selecting a model among a set that may not contain the true model for the purpose of predicting the dependent variable over unseen data. In contrast, BIC is an optimal rule for selecting the true model (or the lowest-dimension model that best describes the data) among a set of models that contains the true model [64].

Note that model selection is ultimately performed by humans, using expert knowledge about the physics and biology of the situation, and with a clear experimental goal (e.g., prediction vs. description). However, when knowledge about the underlying physical or biological phenomena is incomplete, data-driven model selection between plausible models can help researchers gain knowledge about the phenomenon of interest and may point to unconsidered physics.

#### 2.2.8 Practical identifiability analysis

Practical identifiability analysis is performed after model calibration and selection. It utilizes the inferred model and the experimental measurements to find confidence intervals for each inferred parameter. The sparsity of the experimental data set, or large experimental variability can result in practical unidentifiability [12].

There are several methods to conduct practical identifiability analysis of models. A commonly used method calculates the local sensitivities of the model with respect to its parameters to construct the Fisher information matrix (FIM). The method then either uses the eigenvectors of FIM or constructs the correlation matrix to finalize the analysis [65, 66]. Null eigenvectors and high correlations point to the unidentifiable parameters. An alternative, which we use in this work, is profile likelihood-based methods, which are invariant to model reparameterization, are not limited to symmetric confidence intervals, and can even detect structural unidentifiabilities [15].

Profile likelihood-based methods consider one model parameter (e.g., *θ*_*i*_) as fixed at a given value, and then find the MLE of the rest of the parameters (*θ*_*j*_, ∀*j* ≠ *i*) in Eq 15, i.e.,

$$\begin{array}{c} \text{PL}(\theta_{i})=\underset{\theta_{j\neq i}}{\text{min}}\;L\left(\Theta \right). \end{array}$$ (17)

This process is systematically repeated for different values of the fixed model parameter *θ*_*i*_, and then for each parameter *θ*_*j*_ in turn. Then, the confidence interval for each parameter is defined as the set

$$\begin{array}{c} {\text{CI}}_{\text{PL}}(\theta_{i})=\{\theta_{i}|\text{PL}(\theta_{i})\leq L\left(\Theta \right)+\Delta_{\alpha }\} \end{array}$$ (16)

where Δ_*α*_ is the *α*-quantile of the *χ*^2^ distribution with one degree of freedom. A model parameter is deemed as practically identifiable if the corresponding confidence interval is finite.

#### 2.2.9 Goodness of fit

Goodness of fit refers to the assessment of how well the model represents the data using a suitable error metric. In the context of model selection among a set of calibrated models, an unbiased assessment of goodness of fit must rely on hold-out data, i.e., data from the same experimental context (same generating process) but distinct from that used for model calibration and selection. This hold-out data is referred to herein as the validation data set. In this work, we use the mean relative prediction error of the inferred model on the validation dataset as,

$$\begin{array}{c} \text{Error}=\frac{1}{m}\sum_{j=1}^{n_{z}}\sum_{k=1}^{n_{k}}\frac{\sqrt{{\left(Z_{j}\left(t_{k}\right)-z_{j}\left(t_{k},\Theta \right)\right)}^{2}}}{Z_{j}\left(t_{k}\right)} \end{array}$$ (19)

This value predicts the model accuracy when predicting the state variables for any case within the convex hull of the experimental data used to calibrate, select and validate the models.

As an additional metric to assess the accuracy of the model predictions, we calculate the mean relative experimental variance of the data. In other words, this is the standard deviation, among the number of experiment replicates (9), of each observable at each time point, normalized with the value of the mean value of the observable at that time point, and averaged over all time points and observables, i.e.

$$\begin{array}{c} \text{Noise}=\frac{1}{m}\sum_{j=1}^{n_{z}}\sum_{k=1}^{n_{k}}\left(\frac{\sigma_{j}\left(t_{k}\right)}{Z_{j}\left(t_{k}\right)}\right). \end{array}$$ (20)

Note that this value expresses the variance among replicates in normalized terms. This information is critical to properly evaluate the prediction error of the models, which cannot be meaningfully expected to be lower than the intrinsic variability of the experimental data.

#### 2.2.10 Global sensitivity analysis

Global sensitivity analysis (GSA) quantifies the effect that any input has on the system output, averaged over the input domain, i.e., over the hypercube formed by the Cartesian product of the ranges of each input. In the context of the proposed methodology, GSA can be used to rank the model parameters in terms of their quantitative impact on the model predictions. It also is used for identifying unimportant parameters and parameter space regions where each parameter is most important [67]. More importantly, GSA applied to the validated model can allow us to rank the variables that can be controlled during an experiment in terms of their effect on any observable of interest. Whether applied to the model parameters or to the experimentally controllable variables, GSA is an often overlooked and crucial step for both quality assurance and practical application of *in silico* models [35].

Variance-based GSA methods such as Sobol’s [68] and Saltelli’s [69] are the most commonly used in the literature, mainly due to their suitability for non-linear, high-dimensional responses, and for their consideration of interactions between input variables [70]. Variance-based GSA methods are based on a seminal work by Sobol [68], which showed a general decomposition of non-linear, continuous functions (e.g., *Y*_*k*_ in Eq 9) into a set of integrals of increasing dimensionality representing the overall mean, main effects, and interactions of increasing order between variables [71].

$$\begin{array}{c} Y_{k}=f_{0}+\sum_{i=1}^{d}\;f_{i}(X_{i})+\sum_{i<j}^{d}\;f_{ij}(X_{i},X_{j})+\ldots +f_{1,2,\ldots ,d}(X_{1},X_{2},\ldots ,X_{d}) \end{array}$$ (21)

where *f*_0_ is a constant, *f*_*i*_(*X*_*i*_) is the main effect of *X*_*i*_ (effect of only varying *X*_*i*_), and *f*_*ij*_(*X*_*i*_, *X*_*j*_) is the first-order interaction between *X*_*i*_ and *X*_*j*_. Assuming Eq 21 to be square-integrable, the functional decomposition will result in,

$$\begin{array}{c} \text{Var}\left(Y\right)=\sum_{i=1}^{d}V_{i}+\sum_{i<j}^{d}V_{ij}+\ldots +V_{1,2,\ldots ,d} \end{array}$$ (22)

with,

$$\begin{array}{c} V_{i}={\text{Var}}_{X_{i}}(E_{X_{∼i}}(Y|X_{i}))\\ V_{ij}={\text{Var}}_{X_{ij}}(E_{X_{∼ij}}(Y|X_{i},X_{j}))-V_{i}-V_{j} \end{array}$$ (23)

where *X*_∼*i*_ means set of all variables except *X*_*i*_ and E is the expectation operator. Using Sobol’s method, we calculate first and total-order interactions for all the model parameters as,

$$\begin{array}{c} S_{i}=\frac{V_{i}}{\text{Var}(Y)}\\ S_{T_{i}}=\frac{E_{X_{∼i}}({\text{Var}}_{X_{i}}(Y|X_{∼i}))}{\text{Var}(Y)} \end{array}$$ (24)

*S*_*i*_ measures the effect of varying *X*_*i*_ alone averaged over variations in other input parameters, standardized by the total variance. These values are calculated with Monte Carlo sampling, allowing the creation of confidence intervals for the sensitivity indices.

## 3 Results

In this section, we describe the application of the proposed methodology for inferring the population dynamics of the airway-relevant BEAS-2Bs cell line, under different biochemical conditions in a (no flow) static culture environment. The following subsections will illustrate how the methodology is applied, step by step, in the development of a model for BEAS-2B cells and show the challenges and benefits of this strategy. The reader can refer to the Materials and Methods section for a full description of the methodology.

### 3.1 Model proposals

This stage creates a set of model proposals based on the existing knowledge about the specific cell line under study, in this case, BEAS-2B cells. BEAS-2B is a human bronchial epithelial cell line derived from normal, non-cancerous human bronchial tissue closely resembling the primary human bronchial epithelial cell’s morphology and functional characteristics. These cells do not differentiate, and their growth rate is known to be affected by oxygen, glucose, and lactate levels [39, 72].

In a homogeneous, static growth environment with no cell migration or shear stress, Eqs 5 and 6 are reasonable model hypotheses for cell population dynamics of a single cell line that does not differentiate. Also, spatial variations in biochemical concentrations would be negligible, and thus Eq 1 would simplify to Eq 3. The Michaelis-Menten reaction rates for glucose and oxygen (as defined in Eq 8) will yield the initial system of ordinary differential equations (ODEs) as,

$$\begin{array}{cc} \frac{\text{d}n}{\text{d}t}\left(t\right)= & f_{1}(c_{o}\left(t\right))f_{2}(c_{g}\left(t\right))f_{3}(c_{l}\left(t\right))\beta n\left(t\right)(1-\frac{n(t)}{n_{\text{max}}})\\ - & \delta n(t)\\ \frac{\text{d}c_{g}}{\text{d}t}\left(t\right)= & -R_{g}\left(t\right)=-V_{g}n\left(t\right)\frac{c_{g}\left(t\right)}{c_{g}\left(t\right)+{\bar{c}}_{g}}\\ \frac{\text{d}c_{l}}{\text{d}t}\left(t\right)= & -2R_{g}\left(t\right)+\frac{R_{o}\left(t\right)}{3}=\\ & -2V_{g}n\left(t\right)\frac{c_{g}\left(t\right)}{c_{g}\left(t\right)+{\bar{c}}_{g}}+\frac{1}{3}V_{o}n\left(t\right)\frac{c_{o}\left(t\right)}{c_{o}\left(t\right)+{\bar{c}}_{o}} \end{array}$$ (25)

In these equations, lactate production is calculated using the relationship between aerobic respiration and glycolysis [7, 8].

For each of the terms *f*_1_(*c*_*o*_(*t*)) and *f*_2_(*c*_*g*_(*t*)), which account for the potential effect of oxygen and glucose on the cell population growth rate, we select three candidate models, namely zero-order (Eq 6a), first-order (Eq 6b), and MMK with positive feedback (Eq 6c). Similar candidate models are chosen for the lactate effect, *f*_3_(*c*_*l*_(*t*)), but using MMK with negative feedback (Eq 6d) instead. Thus, the model proposal stage results in a total of 27 different candidate models, which will be investigated later in the model inference step via model selection methods.

### 3.2 Structural identifiability analysis

To study the structural identifiability of the models proposed for the BEAS-2B cell line, we focus on the most complex candidate model, i.e., the model with the largest number of parameters and/or most severe non-linearity. The implicit assumption is that, if it is deemed structurally identifiable, then simpler models will be as well. This assumption is reasonable because there are only minor differences between the models being considered.

Global structural identifiability for the model was confirmed using StructuralIdentifiability.jl; an open-source SciML package for structural identifiability analysis [73–75]. In the specific case of the BEAS-2B candidate models proposed in the previous step, the most complex model corresponds to Eq 25 with MMK effects for the three substrates. The observables for performing this analysis are the cell population density and glucose and lactate concentrations. Results showed that this model is structurally identifiable.

### 3.3 Objective function definition

The next step in the methodology is defining the objective function. For the specific case of the BEAS-2B model, we use non-linear least squares (NLS). Let $Z_{j}\in \left\{N_{i},C_{g_{i}},C_{l_{i}}\right\}$ and $z_{i}\in \left\{n_{i},c_{g_{i}},c_{l_{i}}\right\}$, then the least square problem becomes finding the parameters Θ* by,

$$\begin{array}{c} \begin{array}{cc} \Theta^{*}=\text{arg}\;\underset{\Theta }{\text{min}}-\ell \left(\Theta \right)= & \sum_{N_{i}\in S_{T}}w_{N_{i}}{\left(N_{i}-n_{i}\left(\Theta \right)\right)}^{2}\\ + & \sum_{C_{g_{i}}\in S_{T}}w_{G_{i}}{\left(C_{g_{i}}-c_{g_{i}}\left(\Theta \right)\right)}^{2}\\ + & \sum_{C_{l_{i}}\in S_{T}}w_{L_{i}}{\left(C_{l_{i}}-c_{l_{i}}\left(\Theta \right)\right)}^{2} \end{array} \end{array}$$ (26)

where *S*_*T*_ is the training set. As mentioned in Sec. 2.2.3, minimizing Eq 26 is equivalent to using the maximum likelihood estimator in Eq 12 [15] if the experimental data is assumed to have a Gaussian distribution.

### 3.4 Model-based design of experimental protocols

We applied the proposed approach for model-based design of experimental protocols (Sec. 2.2.4) to the inference of a model for cell population dynamics of BEAS-2B cells. Since this is the first mathematical model for BEAS-2Bs, there is no available data or information on which to base decisions about the frequency and resolution required for cell growth experiments, i.e., the number and timing of the experimental measurements required to properly capture the system dynamics. Let us assume that one of the model proposals selected in Sec. 3.1, e.g., Eq 25 with MMK effects, is an appropriate description of the system. We then assume that the model parameters have known values (see supplementary material), which are taken from similar experiments published in the literature for nerve cells [10], osteoblast cells [8], and mesenchymal stromal cells [47].

Using this model with initial conditions consistent with our experimental setup (Sec. 2.1) and assumed model parameters, we generate synthetic data about the observables, namely cell population and concentrations of glucose, lactate, and oxygen as a function of time, for a total of 16 different initial conditions. Gaussian noise was added to the data as described in Sec. 2.2.4.

Solving Eq 26 with the generated synthetic data results in the inferred parameters (Θ*). This process was repeated five times with data sets with different sizes, corresponding to the measurement of the observables at periods of 1, 2, 4, 24, and 48 hours. To compare the performance of the parameter inferences using different sampling periods, we defined error as the difference between the assumed and inferred (Eq 26) parameters,

$$\begin{array}{c} e=\frac{|\Theta -\Theta^{*}|}{\left|\Theta \right|}. \end{array}$$ (27)

**Fig 2:**
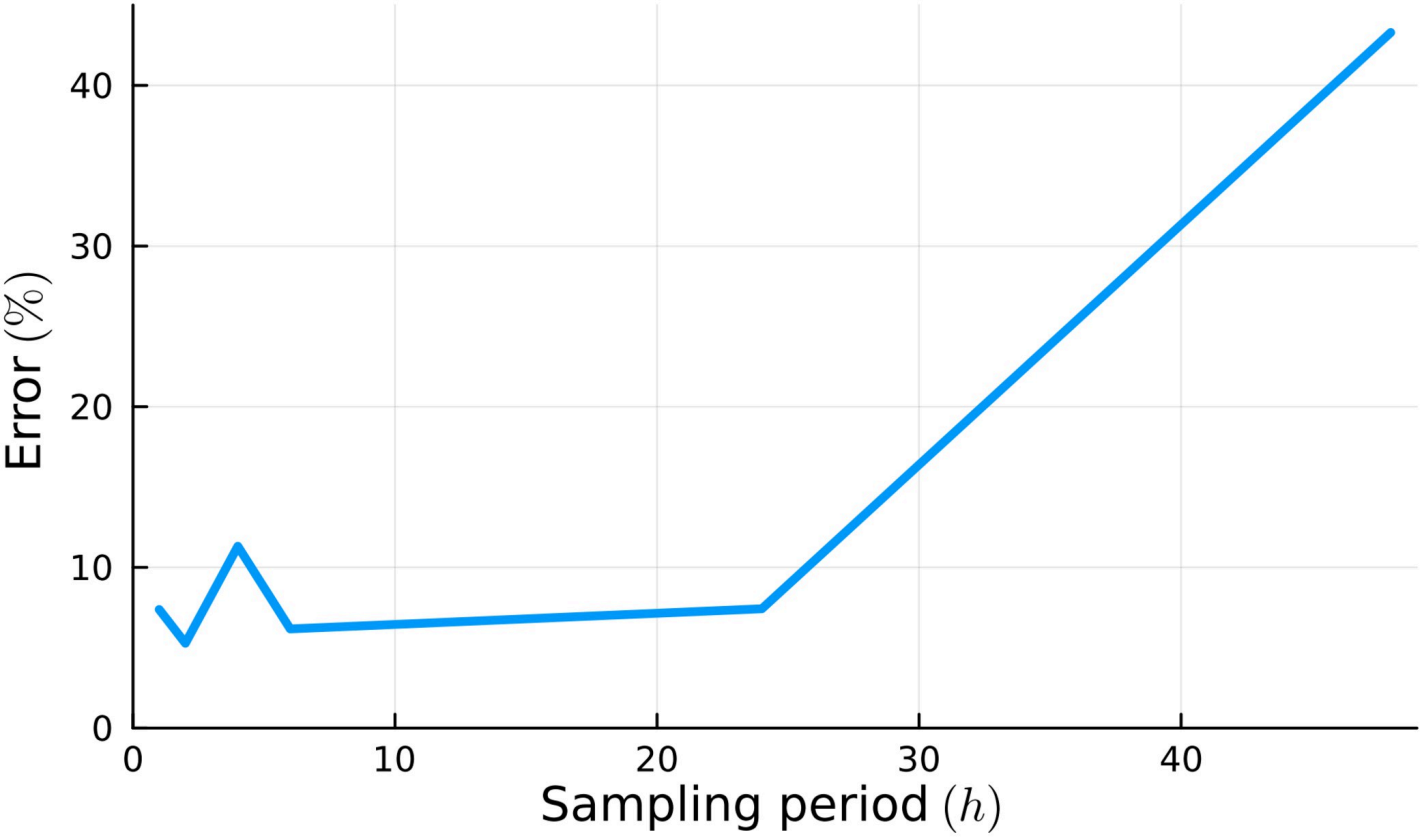
Parameter inference errors for different temporal sampling periods for substrate frequency increases. Parameter inference error decreases as concentration and cell population data is collected more frequently. However, collecting samples at intervals shorter than 24 hrs has no further impact on estimation errors for model parameters.

Fig 2 shows the resulting error in parameter inference as a function of the sampling frequency for the observables. For estimating parameters for the set of model proposals considered here, taking cell population and concentration measurements every 24 hours results in a 7.32% error. Since more frequent measurements do not result in better parameter estimates, we selected 24 hours as the sampling period for our BEAS-2B experiments.

### 3.5 Running and postprocessing the experiments

Experiments were conducted as described in Sec. 2.1. We conducted nine replicates of each experimental condition by doing three runs of each experiment, using three well plates in each run.

Two‐way statistical analysis of variance (ANOVA) test (using the Pingouin [76] Python library) demonstrated statistically significant differences in cell populations between different culture conditions. Specifically, cell populations at time *t* = 102 hours were significantly affected by the change in the culture environment, suggesting that oxygen and initial glucose concentrations affect the growth dynamics. Notably, since oxygen and glucose affect cell population growth, this implies that five of the 27 model proposals can be discarded, since these would make the cell population after *t* = 102 hours independent of oxygen or glucose levels. Hence, only 22 model proposals were considered in the next steps of the methodology.

To prepare the experimental data for model inference, we implemented a data splitting strategy, following best practices in model fitting, selection, and validation typically found in the machine learning literature. The full dataset contains the time evolution of state variables under four different culture environments with four different initial cell densities, giving a total of 16 experimental conditions. This dataset was split into calibration, selection, and validation datasets containing 60%, 20%, and 20% of the data, respectively. Through this process, we implemented a stratified sampling approach to ensure that all experimental conditions were equally represented in each dataset.

### 3.6 Model inference using *in vitro* data

As described in Sec. 2.2.7, using the calibration dataset we inferred the parameters $\left(\beta ,\delta ,V_{g},K_{o},K_{g},K_{l},{\bar{c}}_{g}\right)$ for the 22 candidate models. Due to experimental constraints, it was not possible to measure the oxygen concentration in the growth media, and thus it is assumed to be constant and equal to the oxygen concentration in the environment. We consider this a reasonable assumption, given the time scales involved and the fact that oxygen from the incubator chamber continuously diffuses into the growth media, replenishing the oxygen consumed by the cells. As a result, the advection-diffusion-reaction equation (Eq 1 in Materials and Methods) for oxygen was modified by setting $M_{c_{o}}\left(N\left(t\right),C\left(t\right)\right)=0$, thus indicating a zero rate of change for oxygen concentration. Note, however, that oxygen concentration does affect the cell growth dynamics of BEAS-2B cells. Hence we included *K*_*o*_ in our set of model parameters to be estimated.

Optimization runs were conducted over a search space spanning multiple orders of magnitude, defined as a hypercube of dimension 7 over the range [10^−7^, 10^7^]. A total of 1300 starting points for the optimization were selected as a maxi-min Latin hypercube over the search space to ensure good coverage, for a total of 1300 × 22 = 28600 runs. This process was implemented in the Julia programming language using Flux.jl [77] and Optim.jl [78] libraries for Adam and BFGS optimizers, respectively.

**Fig 3:**
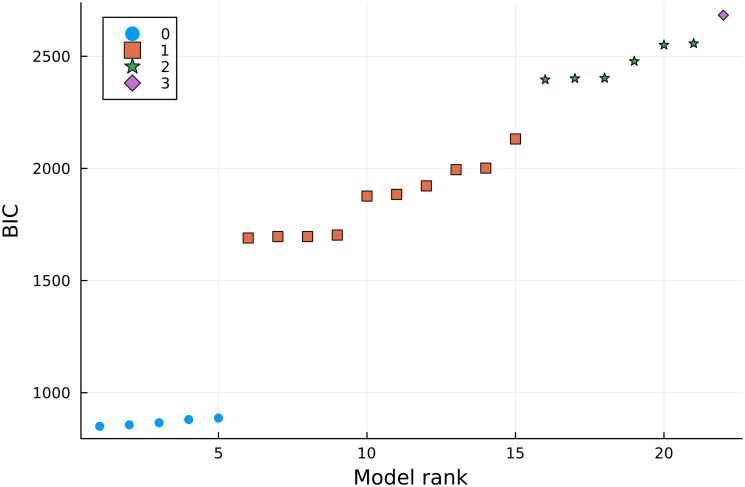
BIC values for the 22 candidate models. Different markers show the number of first-order effects on the cell proliferation rate.

Since parameter calibration is a non-convex multimodal optimization problem with potentially many local optima, we further analyzed the resulting losses and inferred parameters to confirm convergence to a global optimum (S1 Fig in S1 File). These analyses suggest that the model inference does indeed have multiple local optima, but also provide evidence that the optimization strategy used here converges to what is likely the globally optimal model parameters for all 22 candidate models. Once the parameters for all candidate models were inferred with the calibration dataset, we used the selection dataset to calculate the BIC values for all models. Fig 3 shows the resulting BIC values, with different markers/colors indicating the number of first-order modulating effects (Eq 6) present in the model. The BIC criterion strongly suggests that model performance deteriorates as more of the substrates are assumed to have first-order effects on the cell population. Thus, we focused on the top five models (lowest BIC), which include MMK-type effects in at least one of the substrates, namely

$$\begin{array}{cc} F_{\text{env}} & =f_{1}(c_{o}\left(t\right))f_{2}(c_{g}\left(t\right))f_{3}(c_{l}\left(t\right))\\ \text{OxyGluLac} & :\;\;F_{\text{env}}=\frac{c_{o}\left(t\right)}{K_{o}+c_{o}\left(t\right)}\cdot \frac{c_{g}\left(t\right)}{K_{g}+c_{g}\left(t\right)}\cdot \frac{K_{l}}{K_{l}+c_{l}\left(t\right)}\\ \text{GluLac} & :\;\;F_{\text{env}}=1\cdot \frac{c_{g}\left(t\right)}{K_{g}+c_{g}\left(t\right)}\cdot \frac{K_{l}\left(t\right)}{K_{l}+c_{l}\left(t\right)}\\ \text{OxyLac} & :\;\;F_{\text{env}}=\frac{c_{o}\left(t\right)}{K_{o}+c_{o}\left(t\right)}\cdot 1\cdot \frac{K_{l}}{K_{l}+c_{l}\left(t\right)}\\ \text{OxyGlu} & :\;\;F_{\text{env}}=\frac{c_{o}\left(t\right)}{K_{o}+c_{o}\left(t\right)}\cdot \frac{c_{g}\left(t\right)}{K_{g}+c_{g}\left(t\right)}\cdot 1\\ \text{Lac} & :\;\;F_{\text{env}}=1\cdot 1\cdot \frac{K_{l}}{K_{l}+c_{l}\left(t\right)} \end{array}$$ (28)

where the models have been named according to which substrates incorporate an MMK effect.

As seen in Fig 3, the top-performing models have very similar BIC values, so a purely data-driven model selection strategy fails in this case to identify a single winner. However, we note that the model OxyGluLac is mathematically equivalent to the other four models at infinitely large or low values of *K*_*i*_’s, e.g., when *K*_*l*_ → ∞, the OxyGluLac model is equivalent to the OxyGlu model. Hence we select the OxyGluLac model for the remainder of the study, as the best representation of the underlying cell behavior. This choice is also supported by the known biology of BEAS-2B cells.

Table 1 shows the inferred parameters for the OxyGluLac model resulting from the calibration process. Observing the values for *K*_*i*_s, it can be seen that, in the range of the experimental conditions tested in this work,i.e., *c*_*o*_ ∈ [0.05, 0.18] mol m^−3^, *c*_*g*_ ∈ [2, 25] mol m^−3^, and *c*_*l*_ ∈ [2, 20] mol m^−3^, lactate has the highest effect among the biochemical substrates considered. This observation is confirmed later in the Global Sensitivity Analysis step.

**Table 1:** Inferred parameters of the OxyGluLac model using experimental data.

Parameter	Value	(Lower bound, Higher bound)	Unit
*β*	3.83 × 10^−5^	(3.28, 4.42) ×10^−5^	s^−1^
*δ*	3.12 × 10^−5^	(2.60, 3.70) ×10^−5^	s^−1^
*V*_*g*_	8.78 × 10^−19^	(8.23, 9.37) ×10^−19^	s^−1^
*K*_*o*_	1.88 × 10^−5^	(1.58, 7.76) ×10^−5^	mol m^−3^
*K*_*g*_	6.86 × 10^−7^	(5.93, 14.6) ×10^−7^	mol m^−3^
*K*_*l*_	8602	(8600, 86100)	mol m^−3^
${\bar{c}}_{g}$	1.66	(1.31, 2.05)	mol m^−3^
${\bar{c}}_{o}$	6.66×10^−9^		mol m^−3^
*V*_*o*_	2.00×10^−19^		s^−1^

**Fig 4:**
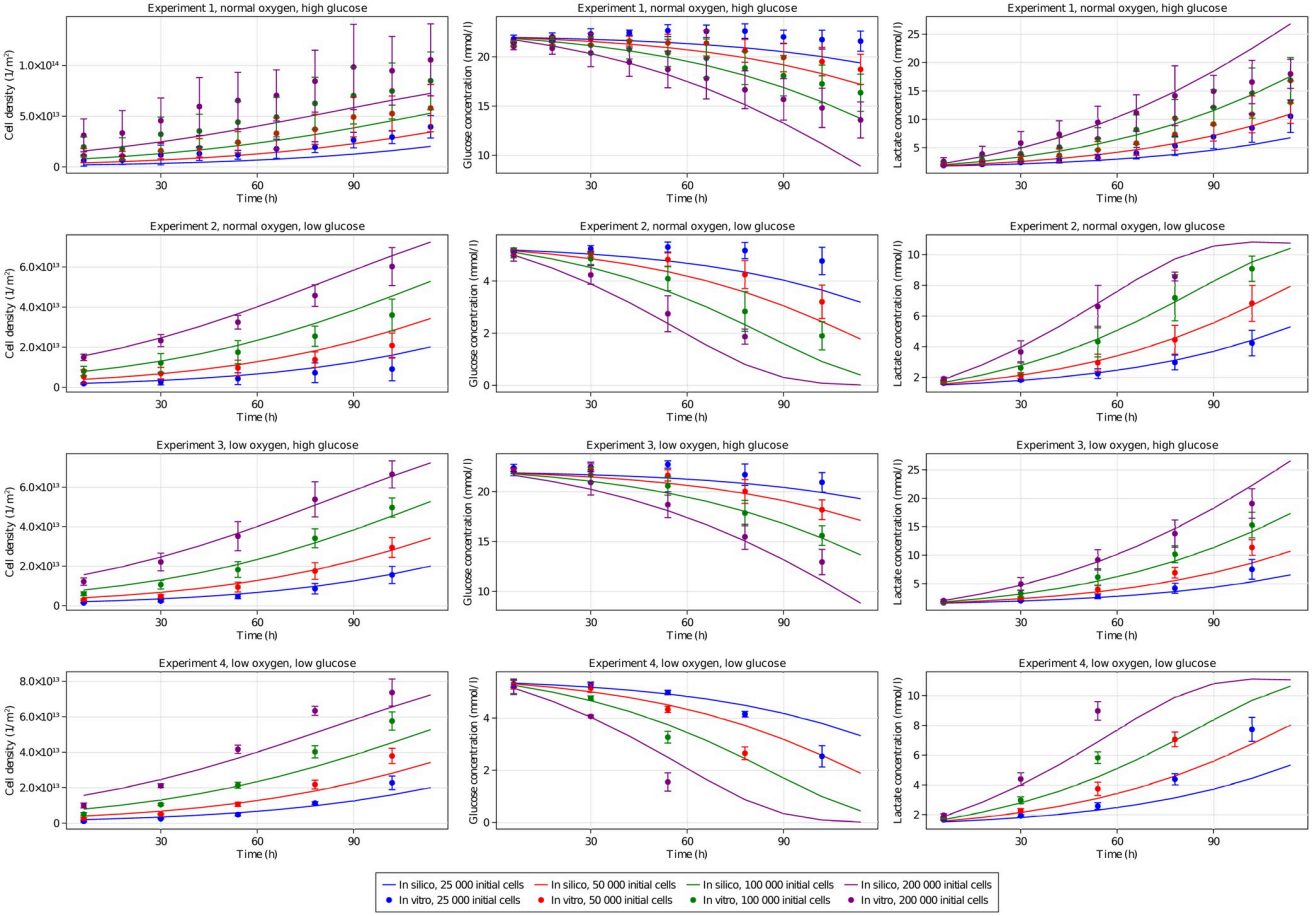
Model inference. Inferred model versus the *in vitro* observations. The *in silico* model results are shown with curves, and the *in vitro* model results are shown as dots with error bars showing standard deviation.

Fig 4 compares the inferred *in silico* model results versus the experimental observations in all experiments. In this figure array, each column in the figure refers to an observable variable (cell density, glucose, and lactate concentrations), and each row represents a different set of experimental conditions (initial concentrations of glucose and oxygen). In each plot, different lines represent different initial cell densities. As the figure illustrates, the calibrated model is indeed able to capture the effect of different biochemical conditions well and accurately predict the resulting cell population through the experiment for all experimental conditions.

Fig 4 also shows the difference between the model prediction and the experimental observations versus the noise of the experimental data, defined as standard deviation over the mean. The figure shows that the model is fairly balanced in underpredicting and overpredicting the state variables. The model predictions are mostly between error bars for the *in vitro* experiments. Perhaps the only exception is the top left subfigure, corresponding to the prediction of cell density under normoxia and high glucose concentration in the culture media. In this case, the model predictions consistently underpredict the experimental data starting at the first time point. These differences are more suggestive of a constant bias than of a prediction error, which can be attributed to systematic errors in our measurements for this specific condition. This observation is supported by the larger dispersion seen in the cell population measurements at the first time point. Further analysis (not shown here for brevity, see S2-S4 Figs in S1 File) confirms that experimental observations are within the confidence intervals of the model predictions.

### 3.7 Practical identifiability analysis

We perform profile likelihood-based practical identifiability analysis using the ProfileLikelihood.jl [79] package. Table 1 shows the resulting confidence intervals for all estimated model parameters are finite, thus proving the practical identifiability of the model based on our experimental data [80].

### 3.8 Goodness of fit

The relative root-mean-square prediction error of the inferred model was calculated using the validation data set as described in Sec. 2.2.9, Eq 19. Results show that the inferred model has a RMSE of 18.3%. For context, the experimental error (noise), calculated as the average of the standard deviations of experimental replicates for each time point and experimental condition, is 18.7%. Based on this, we consider that the model is sufficiently accurate for its applications in support of BEAS-2B tissue engineering.

### 3.9 Global sensitivity analysis

A global sensitivity analysis of the OxyGluLac model was performed. Specifically, we calculated the sensitivity of cell population and glucose and lactate concentrations at time *t* = 114 hr with respect to the experimental conditions that can be controlled, i.e., oxygen concentration in the incubator, initial glucose concentration in the culture media, and initial seeded cell density. For this purpose, we use Sobol’s method (Sec. 2.2.10) with 40,000 Monte Carlo samples. First-order Sobol indices rank the importance of each condition alone, while total-order Sobol indices also include parameter interactions.

**Fig 5:**
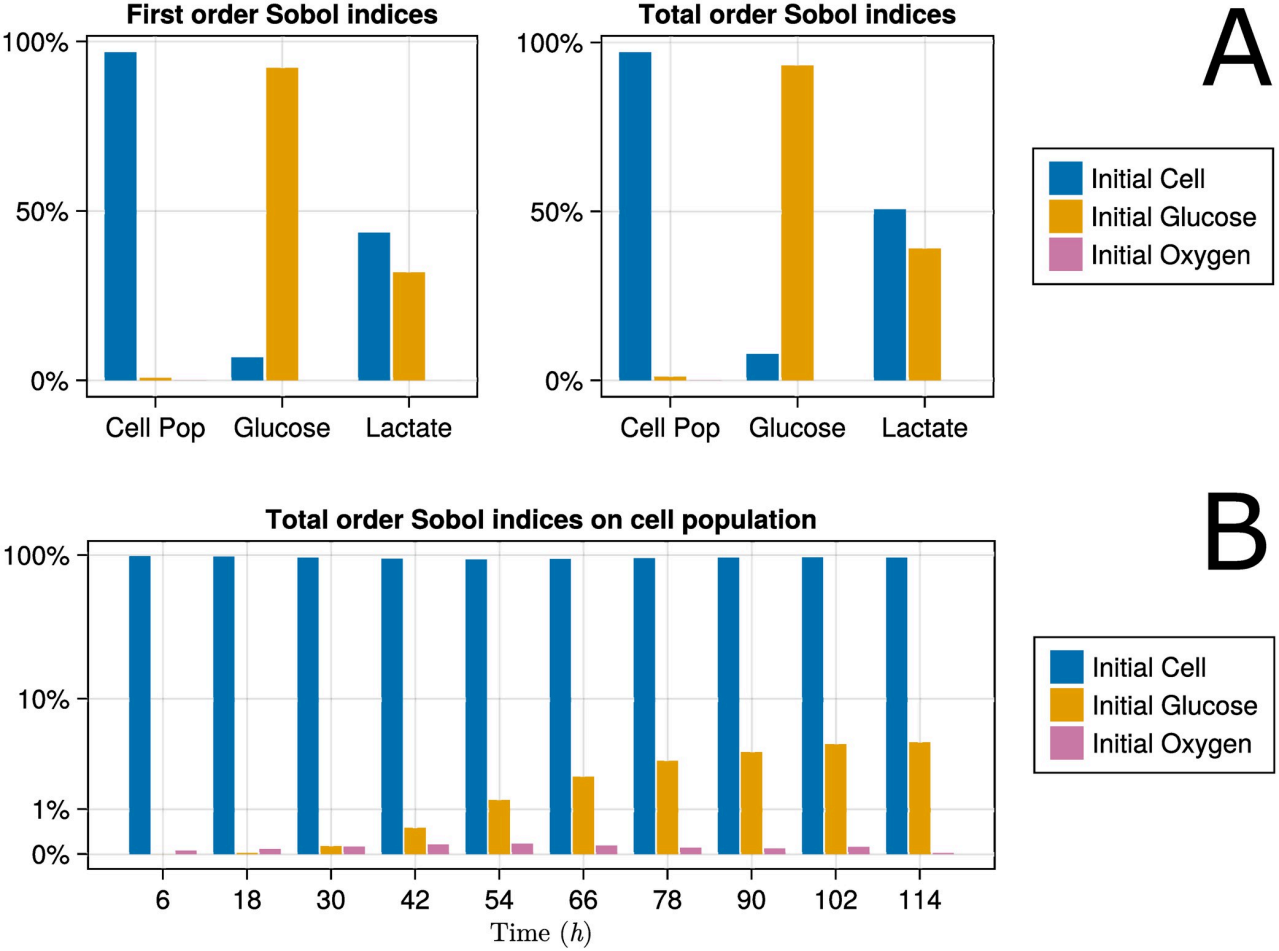
Global sensitivity analysis. A: GSA on terminal values of observables. The x-axis shows the three observables. B: Time evolution of the global sensitivity of cell population to variables controlled during the experiments.

Fig 5A shows the resulting Sobol indices. It can be seen that the initial cell population has the largest effect on both the terminal cell population and lactate concentration while having only a small effect on the terminal glucose concentration. Taken together, these observations suggest that the culture conditions used in the experiments did not impose significant metabolic constraints on the cells during the first *t* = 114 hr. This is also consistent with the lack of significant parameter interactions, as evidenced by the similarity between the first-order and total-order Sobol indices. To further analyze the effect of the culture conditions on the cell population, Fig 5B shows the total-order Sobol sensitivity indices of the cell population throughout the duration of the experiment. It can be observed that the sensitivity increases as the experiment unfolds, suggesting that if the experiment were to be run for longer (e.g., 10 to 15 days), we would see a more significant effect of the biochemical substrates on the cell population.

### 3.10 Application: Optimizing culture conditions

**Fig 6:**
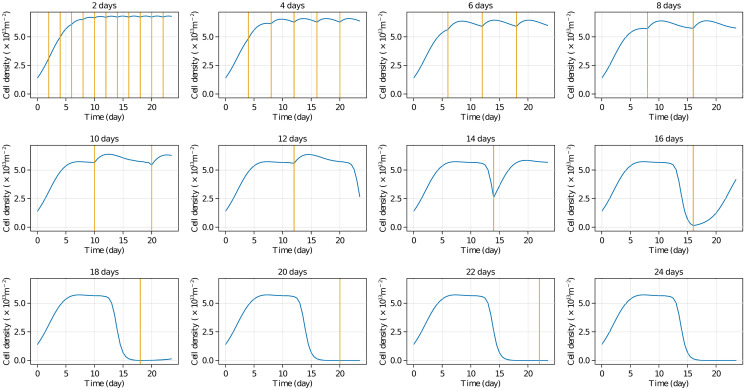
Study of refreshment periods. Lower media refreshment periods result in higher and more stable cell populations.

One application of the SBDO paradigm using our model development methodology is studying the effect of different experimental settings efficiently [7, 81]. Here we study how different media refreshment regimens affect cell population dynamics. *In silico*, this study is implemented by resetting the concentration values of chemical substrates to be equal to their initial values at series of refreshment time points. Fig 6 shows the estimated cell yields after 43 days of culture under different refreshment periods, from 2 to 24 days, indicated as vertical lines. It is observed that refreshing the culture media every 2 to 10 days maintains a relatively stable cell population, with small oscillations that increase in amplitude as the media refreshment period increases. Conducting the experiment with media refreshments every 10 days or more results in drastic decreases in cell population, which become unrecoverable if the media is not refreshed at least every 14 to 16 days. Note that this *in silico* study takes only minutes to run on a desktop computer once an inferred and validated model is available. However, conducting all these experiments *in vitro* would take significant time and incur costs in supplies, equipment, and personnel.

## 4 Discussion

The comprehensive methodology for the development of *in silico* models for neotissue growth proposed in this work leverages state-of-the-art methods for experimental design, data-driven model inference and selection, and post-hoc analysis, validation, and interpretation of biologically informed models based on systems of differential equations.

The proposed methodology addresses the complexity, sparsity, and variability of tissue engineering experiments by implementing specific model formulation calibration, selection, and validation steps that are not typically considered in previous works. For instance, Coy et al. [10] developed a model for the regeneration of nerve cells but did not conduct identifiability and sensitivity analysis, which was used by Villaverde et al. [4]. Our proposed methodology uses both structural and practical identifiability analysis to properly assess the suitability of a model and the ability to infer a unique set of parameters based on the available (noisy) experimental data. In comparison with [4], we implement additional model selection and validation steps based on hold-out data. Furthermore, in contrast with [4, 65], we add an extra step for global sensitivity analysis, which provides important insights for model diagnosis and interpretation [71].

Uniquely, in our methodology, we propose a novel method for model-based design of experimental protocols (MBDEP). Starting from the set of biologically informed model proposals being considered, and leveraging prior information about model parameters, MBDEP optimally selects the spatial and temporal sampling frequency required to minimize the estimation error of the inferred model parameters, taking into account the expected experimental noise. In addition, MBDEP provides valuable information regarding the suitability of the measurement equipment and techniques, vis-a-vis their expected measurement error, for the intended model inference application. This is in contrast with SBDO literature in non-biology related applications, which has focused instead on experimental designs balancing the needs for model inference and optimization. For instance, [82–84] combined simulation models and data-driven surrogate models for the adaptive, sequential design of experiments. In particular, starting from an existing mathematical model of the system, these works determined a set of experimental conditions (i.e., the actual sampling points) required to improve a figure of merit, such as maximizing the information content of the data [85], or optimizing a response variable based on a limited set of computationally expensive *deterministic* computer experiments [82]. In contrast, our proposed model-based experimental protocol design approach aims at determining, *a priori*, the quantity and quality of *noisy* data needed for robust inference of mathematical models for neotissue growth formulated as a system of differential equations. However, once a mathematical model has been inferred for the system, adaptive/sequential sampling methods may be used to gather further data to improve the model, capture additional dynamics, or optimize according to experimental goals.

In the context of our *in vitro* experiments with BEAS-2B cells on well plates, where observables vary in time but not in space, the proposed MBDEP allowed us to adequately capture the time evolution of the observables objectively establishing that measuring cell density every 24 hr was necessary to infer model parameters from experimental data over a wide range of measurement noise. Comparatively, previous work in the literature does not typically provide a rationale for certain aspects of their experimental protocols. For instance, Coy et al. [10] used only the terminal cell density after 24 hr for model inference (a total of 15 measurements), Eleftheriadou et al. [11] used 36 measurements to infer a 16-parameter model, while Duchesne et al. [12] used 10 measurements to infer 6 to 8-parameter model. Future extensions of the BEAS-2B models where spatial variations in cell density and substrate concentrations are present could use the proposed MBDEP to determine the distance between measurements (i.e., spatial sampling frequency) and/or their specific location for robust model inference.

We applied the proposed methodology to develop the first mathematical model to predict population dynamics of BEAS-2B cells *in vitro* under different biochemical environments characterized by glucose, oxygen, and lactate concentrations. Over the validation dataset, the resulting BEAS-2B model exhibited a prediction error of 18.3%, an accuracy comparable to the experimental noise (18.7%), thus suggesting the suitability of the inferred model for the design and optimization of bioreactor devices and experimental protocols to maximize cell yield, reduce variability, improve cell coverage, among others [1, 86].

BEAS-2B cells are widely used as an in vitro platform for numerous studies in airway/lung homeostasis and disease [28, 87–90] spanning drug screening, toxicology, viral cellular response, and more recently in tissue engineering applications focusing on optimization of methods for generating bioengineered lungs [23, 91]. As such, a mathematical model accurately predicting BEAS-2B growth and metabolic activity would be valuable in determining experimental conditions in each context.

As an illustration, the inferred model was used to study the effect of the media refreshment period on the resulting BEAS-2B cell population. It was shown that changing the culture media every 1 to 10 days did not have a significant effect on the final cell population after 43 days. Comparatively, cell culture protocols for BEAS-2B cells and similar airway-relevant tissues typically prescribe media changes every 48 hr to 72 hr in growth media. Insights from experimentally validated *in silico* models may thus translate to significant savings in supplies (e.g., 10X reduction in growth media in this case) and personnel, especially in the context of commercial production of engineered tissues.

### 4.1 Concluding remarks

The *in silico* model considered in this work is based on a system of coupled differential equations describing advection-diffusion-reaction of biochemical substrates and their effect on neotissue growth of a single cell type, without considering multiple cell types and transitions between them. The mathematical model family proposed here can be applied to other single cell line populations with their dynamics affected by glucose, lactate and oxygen concentrations. In such applications, all the model parameters would need to be re-calibrated using relevant experimental data. Also, in the case of biochemical stimuli other than glucose, lactate and oxygen, additional terms representing their rates of change would need to be added to the system of equations, along with their potential effect on the cell proliferation term.

The framework we propose can be easily applied to multiple cell types by adding additional equations similar to Eq 5 for each cell type, and including transitions between cell types through the response terms [92]. Duchesne et al. [12] proposed such a model for the differentiation of chicken erythroid progenitor cells, in which transitions between cell types depended on cell densities only, without considering bio-chemo-mechanical cues. Such formulations could be combined with the proposed methodology for studying the directed differentiation of pluripotent stem cells under different environments [27, 93]. Additional effects such as shear stress [39], scaffold stiffness, and air-liquid interface exposure could be added to the model, e.g., through Eq 6.

*In vitro* neotissue growth, whether under static or perfusion conditions, is a complex multi-scale multi-physics phenomenon [94]. To realize the full potential of SBDO applications in tissue engineering, there is a need for end-to-end *in silico* modeling, including perfusion cell seeding, deposition, attachment, proliferation, migration, and differentiation in response to both biochemical and mechanical cues.

In this context, hybrid Lagrangian-Eulerian formulations that consider scaffold biomechanics, cell-cell, and cell-scaffold interactions [95] while tracking the motion of individual cells or cell parcels within a flow field [39, 96], are promising approaches. Neotissue growth models such as those presented in this work could be combined with hybrid Lagrangian-Eulerian formulations to achieve end-to-end *in silico* neotissue growth modeling.

## Supporting information

S1 File (PDF)

## References

[1] Möller J, Pörtner R. Digital Twins for Tissue Culture Techniques—Concepts, Expectations, and State of the Art. Processes. 2021;9(3):447. doi: 10.3390/pr9030447

[2] Post JN, Loerakker S, Merks RM, Carlier A. Implementing computational modeling in tissue engineering: where disciplines meet. Tissue Engineering Part A. 2022;28(11-12):542–554. doi: 10.1089/ten.tea.2021.0215 35345902

[3] Waters S, Schumacher L, El Haj A. Regenerative medicine meets mathematical modelling: developing symbiotic relationships. NPJ Regenerative Medicine. 2021;6(1):1–8. doi: 10.1038/s41536-021-00134-2 33846347 PMC8042047

[4] Villaverde AF, Pathirana D, Fröhlich F, Hasenauer J, Banga JR. A protocol for dynamic model calibration. Briefings in Bioinformatics. 2022;23(1):bbab387. doi: 10.1093/bib/bbab387 34619769 PMC8769694

[5] Geris L, Guyot Y, Schrooten J, Papantoniou I. In silico regenerative medicine: how computational tools allow regulatory and financial challenges to be addressed in a volatile market. Interface Focus. 2016;6(2):20150105. doi: 10.1098/rsfs.2015.0105 27051516 PMC4759755

[6] Berg M, Holroyd N, Walsh C, West H, Walker-Samuel S, Shipley R. Challenges and opportunities of integrating imaging and mathematical modelling to interrogate biological processes. The International Journal of Biochemistry & Cell Biology. 2022;146:106195. doi: 10.1016/j.biocel.2022.106195 35339913 PMC9693675

[7] Mehrian M, Guyot Y, Papantoniou I, Olofsson S, Sonnaert M, Misener R, et al. Maximizing neotissue growth kinetics in a perfusion bioreactor: An in silico strategy using model reduction and Bayesian optimization. Biotechnology and Bioengineering. 2018;115(3):617–629. doi: 10.1002/bit.26500 29205280

[8] Burova I, Peticone C, De Silva Thompson D, Knowles JC, Wall I, Shipley RJ. A parameterised mathematical model to elucidate osteoblast cell growth in a phosphate-glass microcarrier culture. Journal of Tissue Engineering. 2019;10:2041731419830264. doi: 10.1177/2041731419830264 30858965 PMC6402060

[9] Hossain MS, Bergstrom D, Chen X. Computational modelling of the scaffold-free chondrocyte regeneration: a two-way coupling between the cell growth and local fluid flow and nutrient concentration. Biomechanics and Modeling in Mechanobiology. 2015;14(6):1217–1225. doi: 10.1007/s10237-015-0666-0 25804699

[10] Coy R, Al-Badri G, Kayal C, O’Rourke C, Kingham P, Phillips J, et al. Combining in silico and in vitro models to inform cell seeding strategies in tissue engineering. Journal of the Royal Society Interface. 2020;17(164):20190801. doi: 10.1098/rsif.2019.0801 32208821 PMC7115239

[11] Eleftheriadou D, Berg M, Phillips JB, Shipley RJ. A combined experimental and computational framework to evaluate the behavior of therapeutic cells for peripheral nerve regeneration. Biotechnology and Bioengineering. 2022;119(7):1980–1996. doi: 10.1002/bit.28105 35445744 PMC9323509

[12] Duchesne R, Guillemin A, Crauste F, Gandrillon O. Calibration, selection and identifiability analysis of a mathematical model of the in vitro erythropoiesis in normal and perturbed contexts. In silico biology. 2019;13(1-2):55–69. doi: 10.3233/ISB-190471 31006682 PMC6597985

[13] Zhao F, Melke J, Ito K, van Rietbergen B, Hofmann S. A multiscale computational fluid dynamics approach to simulate the micro-fluidic environment within a tissue engineering scaffold with highly irregular pore geometry. Biomechanics and Modeling in Mechanobiology. 2019;18(6):1965–1977. doi: 10.1007/s10237-019-01188-4 31201621 PMC6825226

[14] Pohlmeyer J, Waters S, Cummings L. Mathematical model of growth factor driven haptotaxis and proliferation in a tissue engineering scaffold. Bulletin of Mathematical Biology. 2013;75(3):393–427. doi: 10.1007/s11538-013-9810-0 23358798

[15] Wieland FG, Hauber AL, Rosenblatt M, Tönsing C, Timmer J. On structural and practical identifiability. Current Opinion in Systems Biology. 2021;. doi: 10.1016/j.coisb.2021.03.005

[16] Miller AJ, Spence JR. In vitro models to study human lung development, disease and homeostasis. Physiology. 2017;32(3):246–260. doi: 10.1152/physiol.00041.2016 28404740 PMC6148341

[17] Tian L, Gao J, Garcia IM, Chen HJ, Castaldi A, Chen YW. Human pluripotent stem cell-derived lung organoids: Potential applications in development and disease modeling. Wiley Interdisciplinary Reviews: Developmental Biology. 2021;10(6):e399. doi: 10.1002/wdev.399 33145915

[18] Van Raemdonck D, Neyrinck A, Cypel M, Keshavjee S. Ex-vivo lung perfusion. Transplant International. 2015;28(6):643–656. doi: 10.1111/tri.12317 24629039

[19] Dhasmana A, Singh A, Rawal S. Biomedical grafts for tracheal tissue repairing and regeneration “Tracheal tissue engineering: An overview”. Journal of Tissue Engineering and Regenerative Medicine. 2020;14(5):653–672. doi: 10.1002/term.3019 32064791

[20] Fishman JM, Wiles K, Lowdell MW, De Coppi P, Elliott MJ, Atala A, et al. Airway tissue engineering: an update. Expert opinion on biological therapy. 2014;14(10):1477–1491. doi: 10.1517/14712598.2014.938631 25102044

[21] Varma R, Poon J, Liao Z, Aitchison JS, Waddell TK, Karoubi G, et al. Planar organization of airway epithelial cell morphology using hydrogel grooves during ciliogenesis fails to induce ciliary alignment. Biomaterials Science. 2022;10(2):396–409. doi: 10.1039/D1BM01327K 34897300

[22] Aoki FG, Varma R, Marin-Araujo AE, Lee H, Soleas JP, Li AH, et al. De-epithelialization of porcine tracheal allografts as an approach for tracheal tissue engineering. Scientific Reports. 2019;9(1):1–12. doi: 10.1038/s41598-019-48450-4 31427611 PMC6700109

[23] Ahmadipour M, Duchesneau P, Taniguchi D, Waddell TK, Karoubi G. Negative Pressure Cell Delivery Augments Recellularization of Decellularized Lungs. Tissue Engineering Part C: Methods. 2021;27(1):1–11. doi: 10.1089/ten.tec.2020.0251 33307958

[24] Haykal S, Salna M, Zhou Y, Marcus P, Fatehi M, Frost G, et al. Double-chamber rotating bioreactor for dynamic perfusion cell seeding of large-segment tracheal allografts: comparison to conventional static methods. Tissue Engineering Part C: Methods. 2014;20(8):681–692. doi: 10.1089/ten.tec.2013.0627 24392662 PMC4115681

[25] Varma R, Soleas JP, Waddell TK, Karoubi G, McGuigan AP. Current strategies and opportunities to manufacture cells for modeling human lungs. Advanced Drug Delivery Reviews. 2020;. doi: 10.1016/j.addr.2020.08.005 32835746 PMC7442933

[26] Varma R, Marin-Araujo AE, Rostami S, Waddell TK, Karoubi G, Haykal S. Pre-Clinical Application of Functional Human Induced Pluripotent Stem Cell-Derived Airway Epithelial Grafts. bioRxiv. 2021;. 10.1002/adhm.202100957 34569180

[27] Takahashi K, Yamanaka S. Induction of pluripotent stem cells from mouse embryonic and adult fibroblast cultures by defined factors. cell. 2006;126(4):663–676. doi: 10.1016/j.cell.2006.07.024 16904174

[28] Han X, Na T, Wu T, Yuan BZ. Human lung epithelial BEAS-2B cells exhibit characteristics of mesenchymal stem cells. Plos one. 2020;15(1):e0227174. doi: 10.1371/journal.pone.0227174 31900469 PMC6941928

[29] Park Yh, Kim D, Dai J, Zhang Z. Human bronchial epithelial BEAS-2B cells, an appropriate in vitro model to study heavy metals induced carcinogenesis. Toxicology and applied pharmacology. 2015;287(3):240–245. doi: 10.1016/j.taap.2015.06.008 26091798 PMC4549192

[30] Garcia-Canton C, Minet E, Anadon A, Meredith C. Metabolic characterization of cell systems used in in vitro toxicology testing: lung cell system BEAS-2B as a working example. Toxicology in Vitro. 2013;27(6):1719–1727. doi: 10.1016/j.tiv.2013.05.001 23669205

[31] Bouquerel C, César W, Barthod L, Arrak S, Battistella A, Gropplero G, et al. Precise and fast control of the dissolved oxygen level for tumor-on-chip. Lab on a Chip. 2022;22(22):4443–4455. doi: 10.1039/D2LC00696K 36314259

[32] Sibinovska N, Žakelj S, Roškar R, Kristan K. Suitability and functional characterization of two Calu-3 cell models for prediction of drug permeability across the airway epithelial barrier. International Journal of Pharmaceutics. 2020;585:119484. doi: 10.1016/j.ijpharm.2020.119484 32485216

[33] Park JY, Ryu H, Lee B, Ha DH, Ahn M, Kim S, et al. Development of a functional airway-on-a-chip by 3D cell printing. Biofabrication. 2018;11(1):015002. doi: 10.1088/1758-5090/aae545 30270851

[34] Duchesne R, Guillemin A, Gandrillon O, Crauste F. Practical identifiability in the frame of nonlinear mixed effects models: the example of the in vitro erythropoiesis. BMC bioinformatics. 2021;22(1):1–21. doi: 10.1186/s12859-021-04373-4 34607573 PMC8489053

[35] Saltelli A, Aleksankina K, Becker W, Fennell P, Ferretti F, Holst N, et al. Why so many published sensitivity analyses are false: A systematic review of sensitivity analysis practices. Environmental modelling & software. 2019;114:29–39. doi: 10.1016/j.envsoft.2019.01.012

[36] Lujan H, Criscitiello MF, Hering AS, Sayes CM. Refining in vitro toxicity models: comparing baseline characteristics of lung cell types. Toxicological Sciences. 2019;168(2):302–314. doi: 10.1093/toxsci/kfz001 30657991

[37] Reddel RR, Ke Y, Gerwin BI, McMenamin MG, Lechner JF, Su RT, et al. Transformation of human bronchial epithelial cells by infection with SV40 or adenovirus-12 SV40 hybrid virus, or transfection via strontium phosphate coprecipitation with a plasmid containing SV40 early region genes. Cancer research. 1988;48(7):1904–1909. 2450641

[38] Haykal S, Soleas JP, Salna M, Hofer SO, Waddell TK. Evaluation of the structural integrity and extracellular matrix components of tracheal allografts following cyclical decellularization techniques: comparison of three protocols. Tissue Engineering Part C: Methods. 2012;18(8):614–623. doi: 10.1089/ten.tec.2011.0579 22332979

[39] Lee H, Marin-Araujo AE, Aoki FG, Haykal S, Waddell TK, Amon CH, et al. Computational fluid dynamics for enhanced tracheal bioreactor design and long-segment graft recellularization. Scientific reports. 2021;11(1):1–14. doi: 10.1038/s41598-020-80841-w 33441927 PMC7807076

[40] Wenger RH, Kurtcuoglu V, Scholz CC, Marti HH, Hoogewijs D. Frequently asked questions in hypoxia research. Hypoxia. 2015;3:35. doi: 10.2147/HP.S92198 27774480 PMC5045069

[41] Banh RS, Iorio C, Marcotte R, Xu Y, Cojocari D, Rahman AA, et al. PTP1B controls non-mitochondrial oxygen consumption by regulating RNF213 to promote tumour survival during hypoxia. Nature cell biology. 2016;18(7):803–813. doi: 10.1038/ncb3376 27323329 PMC4936519

[42] Burova I, Wall I, Shipley RJ. Mathematical and computational models for bone tissue engineering in bioreactor systems. Journal of Tissue Engineering. 2019;10:2041731419827922. doi: 10.1177/2041731419827922 30834100 PMC6391543

[43] Amereh M, Edwards R, Akbari M, Nadler B. In-silico modeling of tumor spheroid formation and growth. Micromachines. 2021;12(7):749. doi: 10.3390/mi12070749 34202262 PMC8303756

[44] Fisher RA. The wave of advance of advantageous genes. Annals of eugenics. 1937;7(4):355–369. doi: 10.1111/j.1469-1809.1937.tb02153.x

[45] Kolmogorov A, Petrovskii I, Piskunov N. A Study of the Diffusion Equation with Increase in the Amount of Substance, and Its Application to a Biological Problem in Selected Works of AN Kolmogorov, vol. 1, 242–270; 1937.

[46] Lagergren JH, Nardini JT, Baker RE, Simpson MJ, Flores KB. Biologically-informed neural networks guide mechanistic modeling from sparse experimental data. PLoS computational biology. 2020;16(12):e1008462. doi: 10.1371/journal.pcbi.1008462 33259472 PMC7732115

[47] Osiecki MJ, McElwain SD, Lott WB. Modelling mesenchymal stromal cell growth in a packed bed bioreactor with a gas permeable wall. PloS one. 2018;13(8). doi: 10.1371/journal.pone.0202079 30148832 PMC6110476

[48] Charlebois DA, Balázsi G. Modeling cell population dynamics. In silico biology. 2019;13(1-2):21–39. doi: 10.3233/ISB-180470 30562900 PMC6598210

[49] Verhulst PF. Notice sur la loi que la population suit dans son accroissement. Corresp Math Phys. 1838;10:113–126.

[50] Simpson MJ, Browning AP, Warne DJ, Maclaren OJ, Baker RE. Parameter identifiability and model selection for sigmoid population growth models. Journal of theoretical biology. 2022;535:110998. doi: 10.1016/j.jtbi.2021.110998 34973274

[51] Hossain MS, Bergstrom D, Chen X. Modelling and simulation of the chondrocyte cell growth, glucose consumption and lactate production within a porous tissue scaffold inside a perfusion bioreactor. Biotechnology Reports. 2015;5:55–62. doi: 10.1016/j.btre.2014.12.002 28626683 PMC5466199

[52] Phipps C, Molavian H, Kohandel M. A microscale mathematical model for metabolic symbiosis: Investigating the effects of metabolic inhibition on ATP turnover in tumors. Journal of Theoretical Biology. 2015;366:103–114. doi: 10.1016/j.jtbi.2014.11.016 25433213

[53] Pohlmeyer J, Cummings L. Cyclic loading of growing tissue in a bioreactor: mathematical model and asymptotic analysis. Bulletin of Mathematical Biology. 2013;75(12):2450–2473. doi: 10.1007/s11538-013-9902-x 24154964

[54] Raue A, Becker V, Klingmüller U, Timmer J. Identifiability and observability analysis for experimental design in nonlinear dynamical models. Chaos: An Interdisciplinary Journal of Nonlinear Science. 2010;20(4):045105. doi: 10.1063/1.3528102 21198117

[55] Bellu G, Saccomani MP, Audoly S, D’Angiò L. DAISY: A new software tool to test global identifiability of biological and physiological systems. Computer methods and programs in biomedicine. 2007;88(1):52–61. doi: 10.1016/j.cmpb.2007.07.002 17707944 PMC2888537

[56] Stoica P, Selen Y. Model-order selection: a review of information criterion rules. IEEE Signal Processing Magazine. 2004;21(4):36–47. doi: 10.1109/MSP.2004.1311138

[57] Arnoud A, Guvenen F, Kleineberg T. Benchmarking global optimizers. National Bureau of Economic Research; 2019.

[58] Bates S, Sienz J, Toropov V. Formulation of the optimal Latin hypercube design of experiments using a permutation genetic algorithm. In: 45th AIAA/ASME/ASCE/AHS/ASC Structures, Structural Dynamics & Materials Conference; 2004. p. 2011.

[59] Urquhart M, Ljungskog E, Sebben S. Surrogate-based optimisation using adaptively scaled radial basis functions. Applied Soft Computing. 2020;88. doi: 10.1016/j.asoc.2019.106050

[60] Kingma DP, Ba J. Adam: A method for stochastic optimization. arXiv preprint arXiv:14126980. 2014;.

[61] Wright S, Nocedal J, et al. Numerical optimization. Springer Science. 1999;35(67-68):7.

[62] Akaike H. Information theory and an extension of the maximum likelihood principle. In: Selected papers of hirotugu akaike. Springer; 1998. p. 199–213.

[63] Schwarz G. Estimating the dimension of a model. The annals of statistics. 1978; p. 461–464.

[64] Chakrabarti A, Ghosh JK. AIC, BIC and recent advances in model selection. Philosophy of statistics. 2011; p. 583–605. doi: 10.1016/B978-0-444-51862-0.50018-6

[65] Daneker M, Zhang Z, Karniadakis GE, Lu L. Systems Biology: Identifiability analysis and parameter identification via systems-biology informed neural networks. arXiv preprint arXiv:220201723. 2022;. 10.1007/978-1-0716-3008-2_4 37074575

[66] Miao H, Xia X, Perelson AS, Wu H. On identifiability of nonlinear ODE models and applications in viral dynamics. SIAM review. 2011;53(1):3–39. doi: 10.1137/090757009 21785515 PMC3140286

[67] Ye M, Hill M. Global sensitivity analysis for uncertain parameters, models, and scenarios. In: Sensitivity analysis in earth observation modelling. Elsevier; 2017. p. 177–210.

[68] Sobol IM. Sensitivity analysis for non-linear mathematical models. Mathematical modelling and computational experiment. 1993;1:407–414.

[69] Saltelli A, Tarantola S, Chan KS. A quantitative model-independent method for global sensitivity analysis of model output. Technometrics. 1999;41(1):39–56. doi: 10.1080/00401706.1999.10485594

[70] Saltelli A, Tarantola S. On the relative importance of input factors in mathematical models: safety assessment for nuclear waste disposal. Journal of the American Statistical Association. 2002;97(459):702–709. doi: 10.1198/016214502388618447

[71] Zhang XY, Trame MN, Lesko LJ, Schmidt S. Sobol sensitivity analysis: a tool to guide the development and evaluation of systems pharmacology models. CPT: pharmacometrics & systems pharmacology. 2015;4(2):69–79. doi: 10.1002/psp4.6 27548289 PMC5006244

[72] Stewart CE, Torr EE, Mohd Jamili NH, Bosquillon C, Sayers I. Evaluation of differentiated human bronchial epithelial cell culture systems for asthma research. Journal of allergy. 2012;2012. doi: 10.1155/2012/943982 22287976 PMC3263641

[73] Dong R, Goodbrake C, Harrington HA, Pogudin G. Computing input-output projections of dynamical models with applications to structural identifiability. arXiv preprint arXiv:211100991. 2021;.

[74] Rackauckas C, Nie Q. Differentialequations.jl–a performant and feature-rich ecosystem for solving differential equations in julia. Journal of Open Research Software. 2017;5(1). doi: 10.5334/jors.151

[75] Bezanson J, Edelman A, Karpinski S, Shah VB. Julia: A fresh approach to numerical computing. SIAM review. 2017;59(1):65–98. doi: 10.1137/141000671

[76] Vallat R. Pingouin: statistics in Python. J Open Source Softw. 2018;3(31):1026. doi: 10.21105/joss.01026

[77] Innes M, Saba E, Fischer K, Gandhi D, Rudilosso MC, Joy NM, et al. Fashionable modelling with flux. arXiv preprint arXiv:181101457. 2018;.

[78] Mogensen PK, Riseth AN. Optim: A mathematical optimization package for Julia. Journal of Open Source Software. 2018;3(24):615. doi: 10.21105/joss.00615

[79] VandenHeuvel DJ. ProfileLikelihood.jl; 2023.

[80] Simpson MJ, Maclaren OJ. A profile likelihood-based workflow for identifiability analysis, estimation, and prediction with mechanistic mathematical models. bioRxiv. 2022; p. 2022–12. 10.1371/journal.pcbi.1011515 PMC10566698 37773942

[81] Mehrian M, Lambrechts T, Papantoniou I, Geris L. Computational modelling of human mesenchymal stromal cell proliferation and extra-cellular matrix production in 3D porous scaffolds in a perfusion bioreactor: The effect of growth factors. Frontiers in Bioengineering and Biotechnology. 2020;8:376. doi: 10.3389/fbioe.2020.00376 32411692 PMC7201129

[82] Jones DR, Schonlau M, Welch WJ. Efficient global optimization of expensive black-box functions. Journal of Global optimization. 1998;13(4):455. doi: 10.1023/A:1008306431147

[83] Sacks J, Welch WJ, Mitchell TJ, Wynn HP. Design and analysis of computer experiments. Statistical science. 1989;4(4):409–423. doi: 10.1214/ss/1177012413

[84] Romero DA, Amon CH, Finger S. Multiresponse metamodeling in simulation-based design applications. Journal of Mechanical Design. 2012;134(9). doi: 10.1115/1.4006996

[85] Shewry MC, Wynn HP. Maximum entropy sampling. Journal of applied statistics. 1987;14(2):165–170. doi: 10.1080/02664768700000020

[86] Geris L, Lambrechts T, Carlier A, Papantoniou I. The future is digital: in silico tissue engineering. Current Opinion in Biomedical Engineering. 2018;6:92–98. doi: 10.1016/j.cobme.2018.04.001

[87] Mirhadi S, Zhang W, Pham NA, Karimzadeh F, Pintilie M, Tong J, et al. Mitochondrial Aconitase ACO2 Links Iron Homeostasis with Tumorigenicity in Non–Small Cell Lung Cancer. Molecular Cancer Research. 2023;21(1):36–50. doi: 10.1158/1541-7786.MCR-22-0163 36214668 PMC9808373

[88] Cao X, Fu M, Bi R, Zheng X, Fu B, Tian S, et al. Cadmium induced BEAS-2B cells apoptosis and mitochondria damage via MAPK signaling pathway. Chemosphere. 2021;263:128346. doi: 10.1016/j.chemosphere.2020.128346 33297271

[89] Costa AJ, Lemes RMR, Bartolomeo CS, Nunes TA, Pereira GC, Oliveira RB, et al. Overexpression of estrogen receptor GPER1 and G1 treatment reduces SARS-CoV-2 infection in BEAS-2B bronchial cells. Molecular and Cellular Endocrinology. 2022;558:111775. doi: 10.1016/j.mce.2022.111775 36096380 PMC9458763

[90] Huang CC, Aronstam RS, Chen DR, Huang YW. Oxidative stress, calcium homeostasis, and altered gene expression in human lung epithelial cells exposed to ZnO nanoparticles. Toxicology in vitro. 2010;24(1):45–55. doi: 10.1016/j.tiv.2009.09.007 19755143

[91] Ahmadipour M, Taniguchi D, Duchesneau P, Aoki FG, Phillips G, Sinderby C, et al. Use of High-Rate Ventilation Results in Enhanced Recellularization of Bioengineered Lung Scaffolds. Tissue Engineering Part C: Methods. 2021;27(12):661–671. doi: 10.1089/ten.tec.2021.0182 34847779

[92] Stiehl T, Marciniak-Czochra A. Characterization of stem cells using mathematical models of multistage cell lineages. Mathematical and Computer Modelling. 2011;53(7-8):1505–1517. doi: 10.1016/j.mcm.2010.03.057

[93] Bluhmki T, Traub S, Müller AK, Bitzer S, Schruf E, Bammert MT, et al. Functional human iPSC-derived alveolar-like cells cultured in a miniaturized 96-Transwell air–liquid interface model. Scientific reports. 2021;11(1):1–19. doi: 10.1038/s41598-021-96565-4 34426605 PMC8382767

[94] Vilanova G, Colominas I, Gomez H. Computational modeling of tumor-induced angiogenesis. Archives of Computational Methods in Engineering. 2017;24(4):1071–1102. doi: 10.1007/s11831-016-9199-7

[95] Decuzzi P, Ferrari M. The adhesive strength of non-spherical particles mediated by specific interactions. Biomaterials. 2006;27(30):5307–5314. doi: 10.1016/j.biomaterials.2006.05.024 16797691

[96] Mardani R. Computational Fluid Dynamics Simulation of Tubular Scaffold Re-cellularization inside Perfusion Bioreactors. University of Toronto (Canada); 2020.

