# Supplementary material for: In silico model development and optimization of in vitro lung cell population growth
Source: PLoS One. 2024 May 15;19(5):e0300902. doi: 10.1371/journal.pone.0300902 (PMC11095723; doi:10.1371/journal.pone.0300902)
Supplement: S1 File — (PDF) [file pone.0300902.s001.pdf]

Supporting information

Inference Results Additional material on the inferred model for BEAS-2Bs population dynamics model.

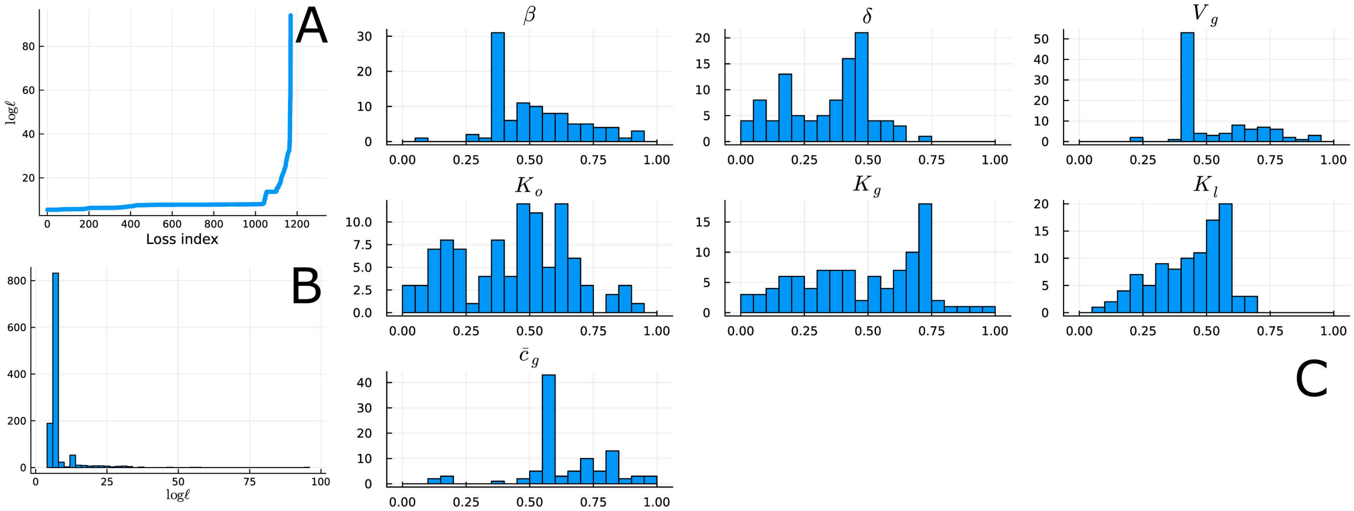

S1 Fig. Convergence analysis of model calibration for the "OxyGluLac" model. A: Histogram of log loss values. B: Waterfall plot for log loss values. Multiple plateaus suggest the existence of multiple local minima. C: Histograms of log) inferred parameter values over the top 100 optimization runs. The presence of multiple modes in all the histograms correlates with the existence of multiple local minima.

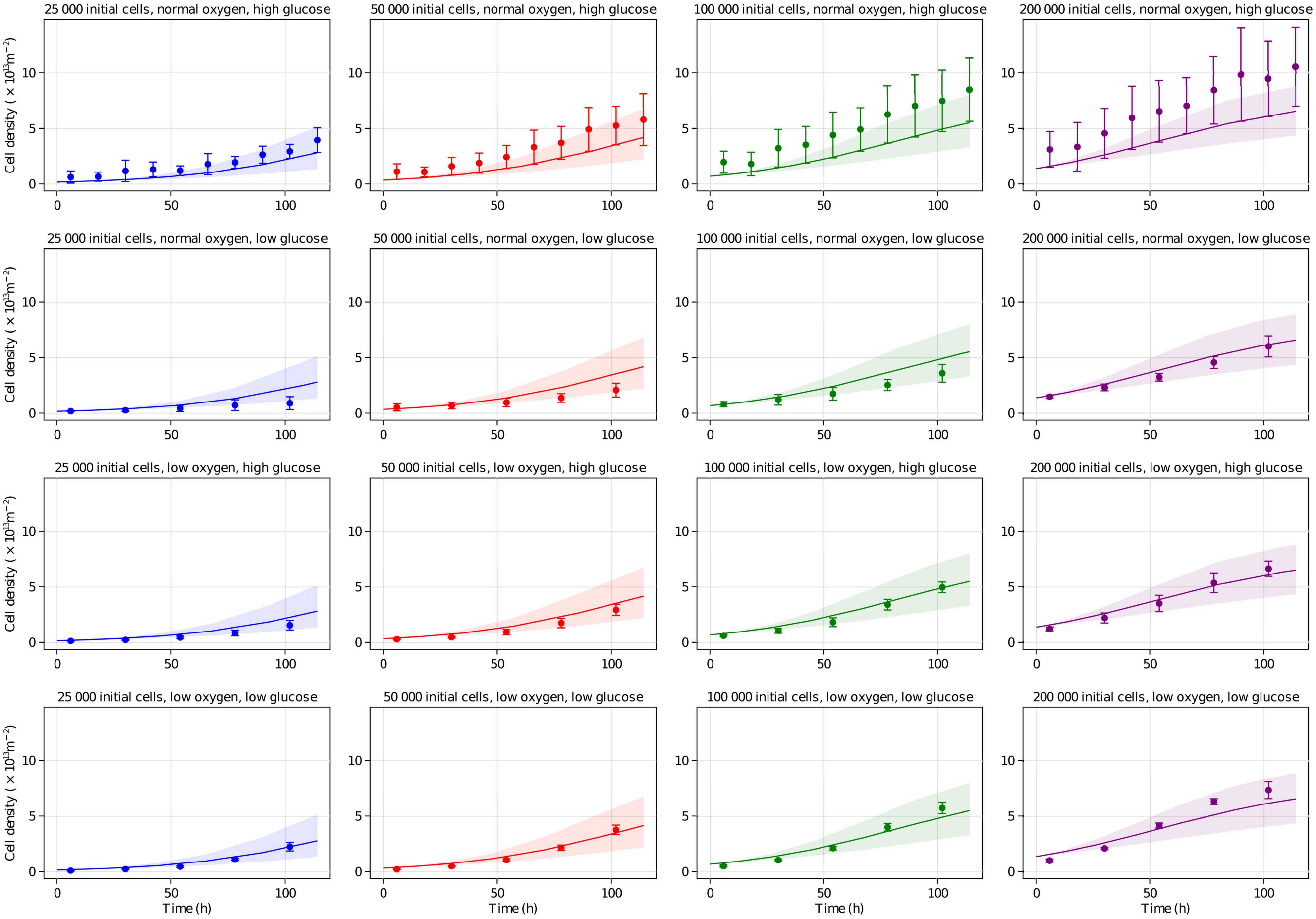

S2 Fig. Population confidence intervals. Inferred model cell populations versus the *in vitro* observations. The *in silico* model results are shown with curves, with the confidence intervals shown using bands, and the *in vitro* model results are shown as dots with error bars showing standard deviation.

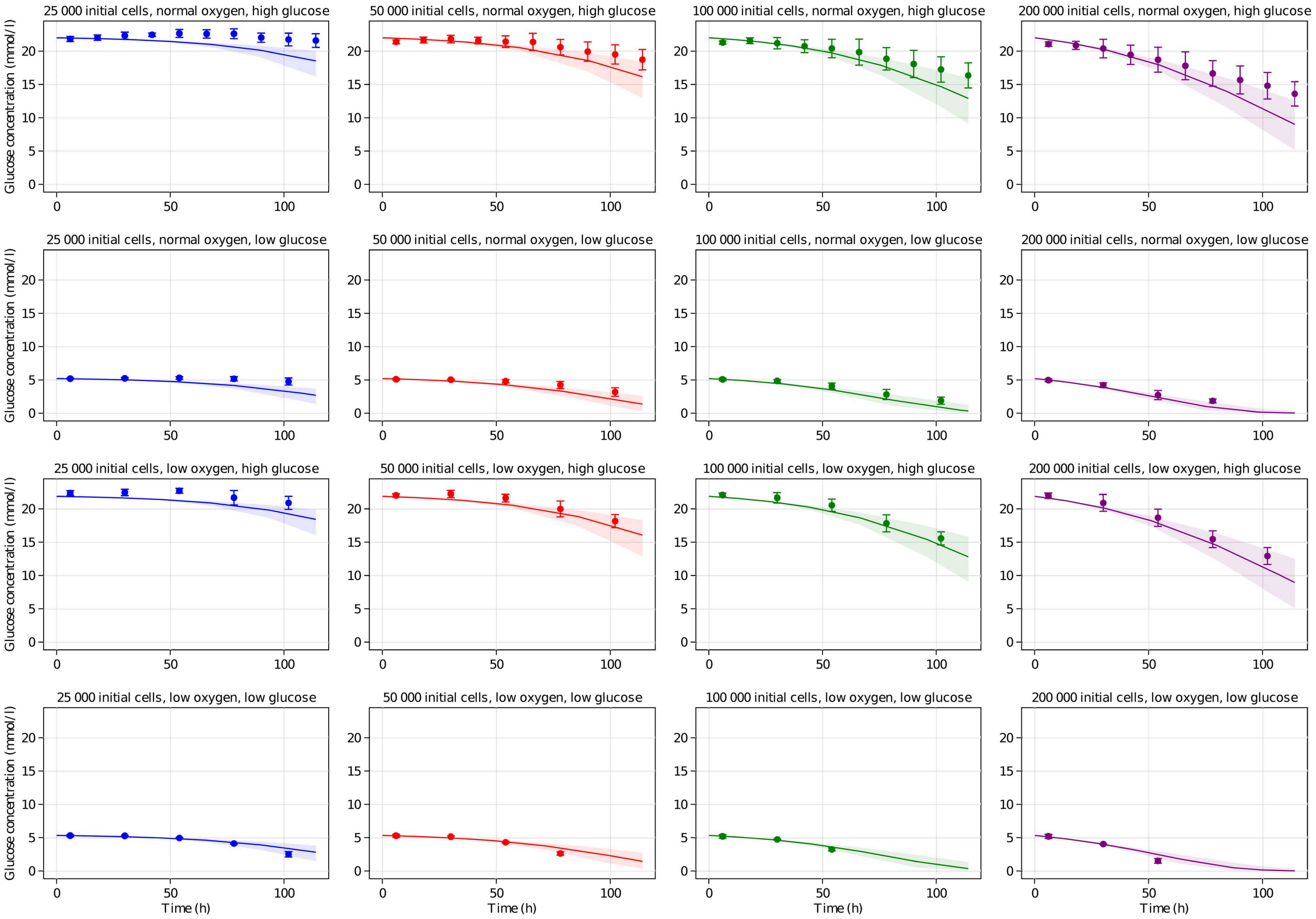

S3 Fig. Glucose confidence intervals. Inferred model glucose concentrations versus the *in vitro* observations. The *in silico* model results are shown with curves, with the confidence intervals shown using bands, and the *in vitro* model results are shown as dots with error bars showing standard deviation.

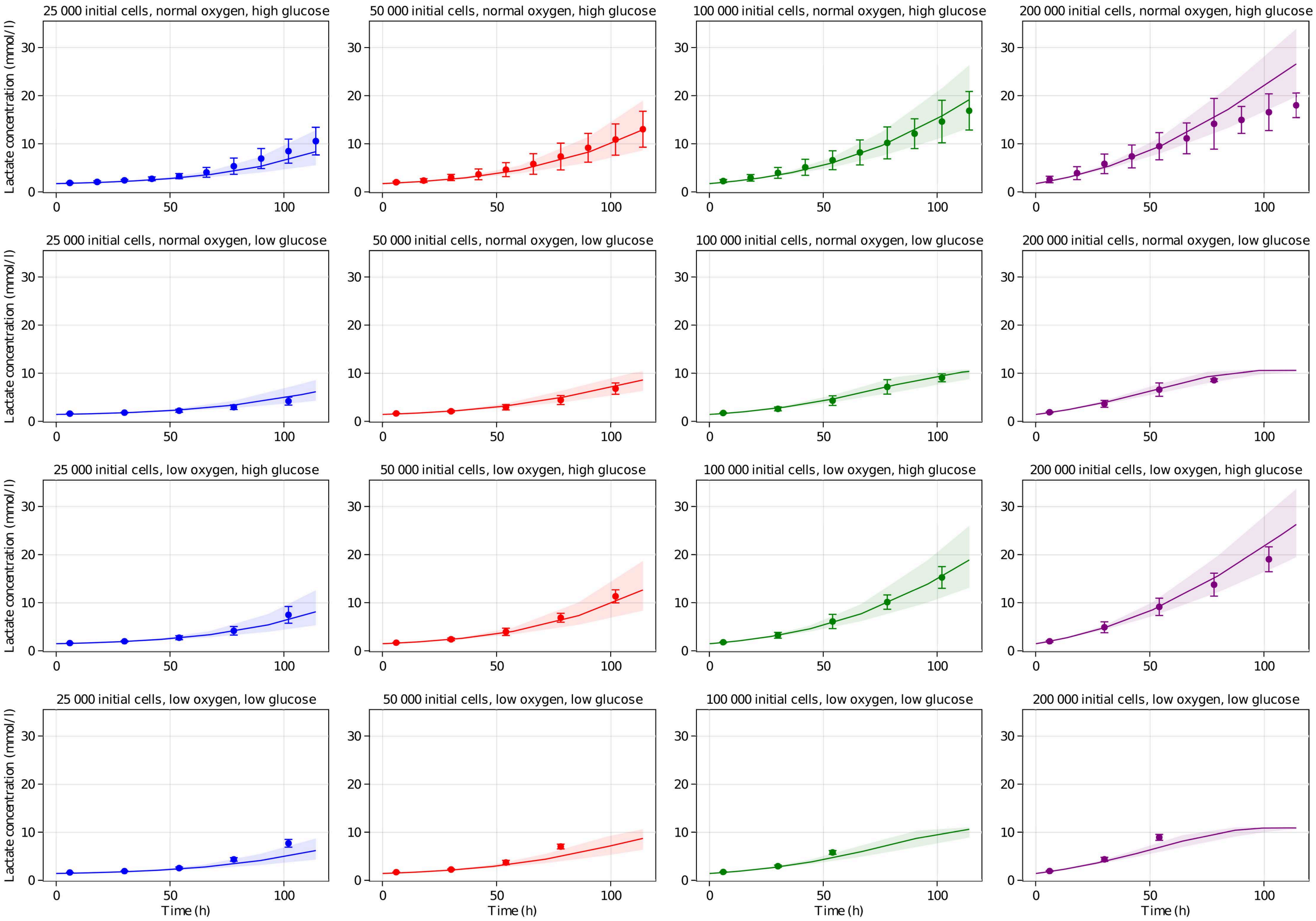

S4 Fig. Lactate confidence intervals. Inferred model lactate concentrations versus the *in vitro* observations. The *in silico* model results are shown with curves, with the confidence intervals shown using bands, and the *in vitro* model results are shown as dots with error bars showing standard deviation.
